# Sorafenib promotes the E3 ubiquitin ligase FBXW7 to increase tau degradation and ameliorate tauopathies

**DOI:** 10.1016/j.apsb.2025.09.024

**Published:** 2025-09-17

**Authors:** Yunqiang Zhou, Yong Wang, Huiying Yang, Chi Zhang, Jian Meng, Lingliang Zhang, Kun Li, Ling-ling Huang, Xian Zhang, Hong Luo, Yunwu Zhang

**Affiliations:** a Fujian Provincial Key Laboratory of Neurodegenerative Disease and Aging Research, Institute of Neuroscience, School of Medicine, Xiamen University, Xiamen 361102, China; b Xiamen Key Laboratory of Brain Center, The First Affiliated Hospital of Xiamen University, School of Medicine, Xiamen University, Xiamen 361102, China

**Keywords:** Alzheimer's disease, FBXW7, Sorafenib, Tau, Tauopathy, Ubiquitin-proteasome pathway

## Abstract

Tauopathies, including Alzheimer's disease (AD), are a series of neurodegenerative diseases characterized by pathological accumulation of the microtubule-associated protein tau. Since the abnormal modification and deposition of tau in nerve cells are crucial for tauopathy etiology, methods for reducing tau levels, such as promoting tau degradation, may become effective strategies for disease treatment. Herein, we identified that sorafenib significantly reduced total tau and phosphorylated tau levels through screening FDA-approved drugs. We showed that sorafenib treatment attenuated cognitive deficits and tau pathologies in PS19 tauopathy model mice. Mechanistically, we found that sorafenib inhibited multiple kinases involved in tau phosphorylation and promoted autophagy. Importantly, we further demonstrated that sorafenib also promoted the expression of the E3 ubiquitin ligase FBXW7, which could bind tau and mediate tau degradation through the ubiquitin–proteasome pathway. Finally, we showed that FBXW7 expression decreased in the brains of AD patients and tauopathy model mice, and that overexpression of FBXW7 in the hippocampus attenuated cognitive deficits and tau pathologies in PS19 mice. These results suggest that sorafenib may be a promising treatment option for tauopathies by promoting tau degradation and reducing tau phosphorylation, and that targeting FBXW7 could also serve as an alternative therapeutic strategy for tauopathies.

## Graphical abstract

Sorafenib mitigates tauopathy *via* multiple pathways: sorafenib inhibits various kinases to reduce tau phosphorylation, enhances autophagy-mediated tau degradation, and upregulates FBXW7 to promote the ubiquitin-proteasome degradation of ptau231.

**Figure:**
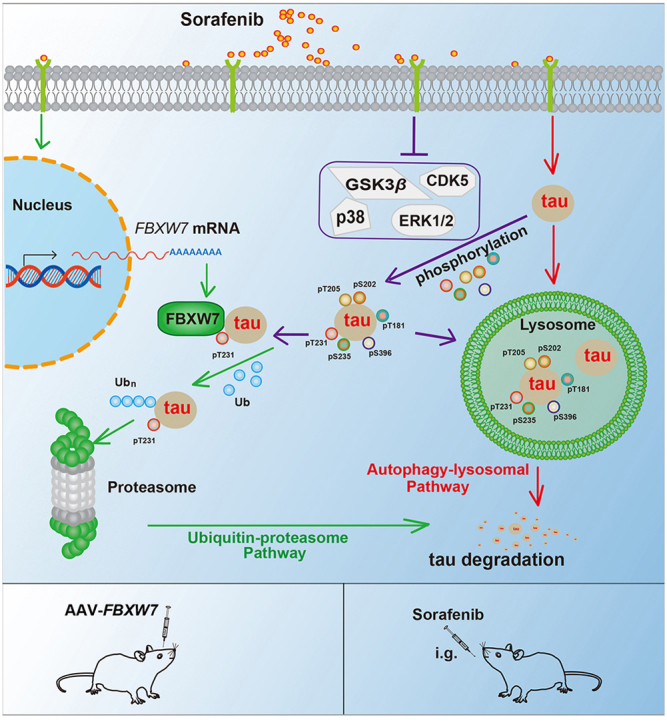

## 1 Introduction

Tauopathy refers to a group of neurodegenerative diseases characterized by the abnormal deposition of the microtubule-associated protein tau in the brain, of which Alzheimer's disease (AD) is the most common form^1^^,^^2^. AD exhibits two major pathological hallmarks in the brain: extracellular senile plaques composed of amyloid beta (A*β*) peptides and intracellular neurofibrillary tangles composed of hyperphosphorylated tau^3^. Tau phosphorylation in the brains of AD patients is markedly elevated, being at least three to four times higher than in aging brains without dementia^4^. Research indicates that tau plays a pivotal role in the etiology of AD and other tauopathies, as the accumulation of hyperphosphorylated tau contributes to synaptic dysfunction and cognitive decline^3^. Therefore, enhancing the degradation of tau, particularly the elimination of hyperphosphorylated tau, may become a crucial strategy for disease intervention^2^^,^^5^^,^^6^.

Sorafenib is a small-molecule tyrosine kinase inhibitor that has been used for various cancer treatments^7^, ^8^, ^9^, ^10^. Several previous studies have shown that sorafenib treatment has protective effects in AD model mice with A*β* pathologies. For example, a 3-day sorafenib treatment suppressed A*β*-mediated astrogliosis in 5xFAD mice^11^. Chronic sorafenib treatment also improved working memory in elderly APPswe mice by lowering NF-*κ*B, COX-2, and iNOS expression, thereby reducing neuroinflammation^12^. However, although sorafenib is a multitargeted tyrosine kinase inhibitor^9^^,^^10^ and may affect the activity of kinases phosphorylating tau, whether and how sorafenib exerts protection against tau pathologies remains unknown.

Herein, we screened 54 U.S. Food and Drug Administration (FDA)-approved small-molecule drugs that can cross the blood–brain barrier in tau-expressing cells and identified sorafenib as a potent drug for reducing total tau and phosphorylated tau levels. We demonstrated that sorafenib can significantly ameliorate cognitive deficits and tau pathologies in PS19 tauopathy model mice through multiple mechanisms, including promoting the E3 ubiquitin ligase FBXW7 to facilitate the degradation of tau and phosphorylated tau. Finally, we showed that overexpression of FBXW7 also attenuated cognitive deficits and tau pathologies in PS19 mice.

## 2 Materials and methods

### 2.1 Cell cultures and treatments

HEK293T and SH-SY5Y cells were cultured in high-sugar Dulbecco's modified Eagle's medium (DMEM, ThermoFisher Scientific, Waltham, MA, USA) supplemented with 10% fetal bovine serum (FBS, ThermoFisher Scientific) and 1% Penicillin–Streptomycin (ThermoFisher Scientific). HEK293 cells stably expressing tau (HEK293-tau) were kindly provided by Dr. Jianzhi Wang, and cultured in medium containing 45% high-sugar DMEM, 45% Opti-MEM medium (ThermoFisher Scientific), 10% FBS, 1% Penicillin–Streptomycin, and 200 μg/mL G418 (ThermoFisher Scientific)^13^. Primary neurons were isolated from the brains of postnatal Day 0 (P0) mice following established protocols described previously^14^, and cultured in Neurobasal medium (ThermoFisher Scientific) supplemented with 1% glutamine (ThermoFisher Scientific), 2% B27 (ThermoFisher Scientific), and 1% Penicillin–Streptomycin. Half of the medium was changed every other day.

For generating SH-SY5Y cells with stable tau(P301L)-EGFP expression, we first packaged lentiviruses by transfecting the tau(P301L)-EGFP plasmid and other viral elements (pMDL, pVSVG, and pRev) using Polyethyleneimine (LABLEAD Inc., Beijing, China), following the manufacturer's instructions. Lentiviruses secreted in the media were collected through centrifugation and stored at −80 °C for future use. For optimal infection, SH-SY5Y cells were kept at 30%–40% confluency, and DMEM containing polybrene (10 μg/mL, Beyotime, Shanghai, China) was added prior to lentivirus infection. 48 h after infection, SH-SY5Y cells were subjected to fluorescence-activated cell sorting. Single cells with tau(P301L)-EGFP expression were expanded and cultured using the SH-SY5Y cell culture conditions.

### 2.2 Plasmids

A full-length human tau with the P301L mutant, fused with the EGFP tag, was constructed in the CD513B-1 vector without puromycin resistance. Full-length human tau and its mutant plasmids (T181A and T231A) were constructed using a pcDNA3.1-myc vector. A full-length human FBXW7 plasmid was constructed using a pcDNA3.3-Flag vector. *FBXW7*-targeting shRNAs were cloned into a Plko.1 vector. Their sequences are: scrambled negative control *shRNA*: 5′-TATGTCAAGTTGTATAGTTA-3′; *FBXW7 shRNA#1*: 5′-ATGGGTTTCTACGGCACATTA-3′; *FBXW7 shRNA#2*: 5′-TGCCTGAGCATGTCCACATTA-3′.

### 2.3 Drug screening

SH-SY5Y cells with tau(P301L)-EGFP expression were treated with 10 μmol/L of 54 FDA-approved drugs (Targetmol, Shanghai, China) that can cross the blood-brain barrier or the DMSO vehicle control for 24 h. Equal amounts of cell lysates were analyzed by immunoblotting for total tau and tau phosphorylated at Serine 404 (pS404) levels.

### 2.4 Antibodies

Antibodies used in the present study including anti-FBXW7 (A5872, Abclonal, Wuhan, China), anti-FBXW7 (55290-1-AP, Proteintech, Wuhan, China), anti-total tau (tau5; AHB0042, Thermo Fisher Scientific), anti-tau pS199 (44734G, Thermo Fisher Scientific), anti-tau pS202/T205 (AT8; MN1020, Thermo Fisher Scientific), anti-tau pS396 (44752G, Thermo Fisher Scientific), anti-tau pT231 (A19710, Abclonal), anti-tau pT231/S235) (20473, Cell Signaling Technology, Danvers, MA, USA), anti-tau pT181 (12885, Cell Signaling Technology), anti-tau pS404 (20194, Cell Signaling Technology), anti-tau pS202 (11834, Cell Signaling Technology), anti-tau pS235 (R25855, Zen Bio, Chengdu, China), anti-LC3 (3868S, Cell Signaling Technology), anti-mTOR (2983, Cell Signaling Technology), anti-p-mTOR (Ser2448) (5536, Cell Signaling Technology), anti-S6 (2217, Cell Signaling Technology), anti-pS6 (5364, Cell Signaling Technology), anti-ERK1/2 (4695, Cell Signaling Technology), anti-pERK1/2 (4370, Cell Signaling Technology), anti-GSK3*β* (51065-1-AP, Proteintech), anti-pGSK3*β* (Y216, Y279) (44604G, Thermo Fisher Scientific), anti-p38 (9212, Cell Signaling Technology), anti-p-p38 (4511, Cell Signaling Technology), anti-CDK5 (sc-173, Santa Cruz Biotechnology, Dallas, TX, USA), anti-GFP (50430-2-AP, Proteintech), anti-flag (20543-1-AP, Proteintech), anti-flag (66008-4-Ig, Proteintech), anti-myc (2276S, Cell Signaling Technology), anti-myc (16286-1-AP, Proteintech), anti-HA (51064-2-AP, Proteintech), anti-Ub (PTM-1106RM, PTM biolabs, Hangzhou, China), anti-GFAP (3670, Cell Signaling Technology), anti-Tuj1 (sc-173, Biolegend, San Diego, CA, USA), anti-NeuN (94403, Cell Signaling Technology), anti-Iba1 (17198, Cell Signaling Technology), anti-GAPDH (ab0038, Abways, Shanghai, China), and anti-*β*-actin (AC026, Abclonal). Horseradish peroxidase-conjugated secondary antibodies were from ThermoFisher Scientific (31430 and 31460).

### 2.5 Immunoblotting and immunoprecipitation

Cells or tissue samples were lysed with 1% TNEN (20 mmol/L tris-HCl, pH 8.0, 100 mmol/L NaCl, 1 mmol/L ethylenediaminetetraacetic acid, and 0.5% NP-40, supplemented with protease and phosphatase inhibitors). Equal amounts of protein lysates were subjected to SDS-PAGE and immunoblotting. Protein bands were detected using the Chemiluminescence Imaging System Azure 300 instrument (Azure Biosystems, Dublin, CA, USA). For immunoprecipitation, 1 mg of protein lysate was incubated with appropriate antibodies or IgG, together with Protein G-Agarose beads (Yeasen Biotechnology, Shanghai, China) overnight at 4 °C. Immunoprecipitated proteins were then analyzed by immunoblotting. Protein band intensity was quantified using the ImageJ software (National Institutes of Health, Bethesda, MD, USA).

### 2.6 CCK-8 assay

Cells were seeded in 96-well plates and then treated with different concentrations of drugs for different periods. Cell viability was assessed using a CCK-8 kit (CA1210; Solarbio, Beijing, China) according to the manufacturer's protocols.

### 2.7 Single-molecule immunoassay

The levels of tau pT231, pT217, and pT181 in sorafenib-treated cells were analyzed on a fully automatic single-molecule detector (AST-Sc-Lite; AstraBio, Suzhou, China)^15^. Briefly, 25 μL samples were mixed with magnetic beads coated with a capture antibody in the protective reagent and incubated for 6 min. A single-molecule fluorescently labeled detection antibody was added, mixed well, and incubated at 40 °C for another 4 min. The magnetic beads in the mixture were transferred to a flow cell by magnetic adsorption to wash and remove unlabeled fluorophores. The fluorescence image was taken with an integrated fluorescence microscope. The single molecule signal was analyzed and converted by a Sc-Lite analyzer, and the protein concentration was calculated through a pre-prepared standard curve.

### 2.8 Animals

PS19 mice (IMSR_JAX:008169, Jackson Laboratory, Bar Harbor, ME, USA) and their wild-type (WT) littermate controls in the C57BL/6J background were housed in a specific pathogen-free facility under a 12 h light–dark cycle, with *ad libitum* access to food and water. Animal procedures followed the guidelines of the National Institutes of Health Guide for the Care and Use of Laboratory Animals, and were approved by the Animal Ethics Committee of Xiamen University (XMULAC20220202).

### 2.9 Human samples

Human brain cortical tissue lysates were obtained through Material Transfer Agreement from National Health and Disease Human Brain Tissue Resource Center and Neurodegenerative Disorder Research Center in China. Subject information (neuropathological diagnosis, death age, and gender) is provided in Supporting Information Table S1. All human subjects gave written informed consent before sample collection.

### 2.10 Pharmacological treatments

7-Month-old PS19 male mice and their littermate male WT controls were treated with sorafenib (MedChemExpress, Monmouth Junction, NJ, USA) for 2 months before behavioral analysis. During behavioral analysis, mice were kept under treatment. Sorafenib was dissolved in 20% HP-*β*-CD with 2.5% DMSO, adjusted to a concentration of 10 mg/kg, and administered to mice by intragastric gavage every other day.

For treatment in cultured cells and neurons, sorafenib was prepared in DMSO at 10 mmol/L and diluted 1:1000 to a final concentration of 10 μmol/L with 0.1% DMSO. In some experiments, 10 μmol/L of the proteasomal inhibitor MG132 (MedChemExpress) or 10 μmol/L of the lysosomal inhibitor chloroquine (MedChemExpress) was used together with sorafenib.

### 2.11 Adeno-associated virus (AAV) infection

AAV2/9 (serotype 2/9) viruses expressing FBXW7 or a scrambled control were packaged by OBIO Technology (Shanghai, China). For *in vivo* injection, one microliter of AAV (2.5 × 10^12^ V G./mL) containing FBXW7 or the control was slowly administered into the CA1 region of the bilateral hippocampus of 5-month-old PS19 male mice and littermate controls under hypothermic anesthesia.

### 2.12 Immunofluorescence

Treated cells were fixed in 4% paraformaldehyde, permeabilized in 0.5% Triton X-100, and blocked in 5% BSA at room temperature for 1 h. Cells were then incubated with appropriate primary antibodies overnight at 4 °C, followed by incubation with the corresponding fluorescently conjugated secondary antibody and DAPI at room temperature for 1 h. After mounting, cell images were captured using a DV1000 MPE-B confocal microscope (Olympus, Tokyo, Japan).

### 2.13 Quantitative real-time PCR (qRT-PCR)

Total RNAs were extracted using TRIzol reagent (ThermoFisher Scientific). cDNA synthesis was performed with the HiScript III All-in-one RT SuperMix Perfect (Vazyme, Nanjing, China). qRT-PCR was then carried out on a LightCycler 480 Real-Time PCR System (Roche Applied Science, Shanghai, China) using the SYBR GREEN QPCR KIT (Abclonal). The sequences of the primers used are as follows:

for human *FBW7*, forward: 5′-CGAACTCCAGTAGTATTGTGGACCT-3′;

reverse: 5′-TTCTTTTCATTTTTGTTGTTTTTGTATAGA-3′;

for mouse *Fbxw7*, forward: 5′-GCCTGAGCATGTCCACGTTA-3′;

reverse: 5′-ACTTCAGAACCATGGTCCAACTT-3′;

for mouse *β*-actin, forward: 5′-GGCTGTATTCCCCTCCATCG-3′;

reverse: 5′-CCAGTTGGTAACAATGCCATGT-3′;

for human *β-*actin, forward: 5′-CATGTACGTTGCTATCCAGGC-3′;

reverse: 5′-CTCCTTAATGTCACGCACGAT-3′.

### 2.14 Behavioral analysis

Treated mice were subjected to behavioral tests including the open-field test, the novel object recognition test, the nest-building test, the Morris water maze test, and the three-chamber social interaction test, following protocols previously reported^16^. Mice were handled by the experimenter three days before the start of behavioral tests to allow them to acclimate. 30 min prior to each experiment, mice were transferred to the testing room for acclimation to the surrounding environment and lighting. All behavioral analyses were conducted in a double-blinded manner. An intelligent video tracking software (Panlab, Harvard Apparatus, Holliston, MA, USA) was used for data collection and analysis.

### 2.15 Statistical analysis

All experiments were performed in three or more independent sample replicates, and statistical analysis was performed using GraphPad Prism 8.3 software (GraphPad Software, Boston, MA, USA). Student's *t*-test (two-tailed distribution) was used for differences between the two groups, and one-way ANOVA or two-way ANOVA was used for multiple comparisons between more than two groups. All data are presented as mean ± standard error of mean (SEM). *P* < 0.05 was considered to be statistically significant.

## 3 Results

### 3.1 Identification of sorafenib as a promising drug for reducing tau levels

To mimic tau pathology, we first generated a construct containing the full-length human tau isoform fused with EGFP at the carboxyl terminus and carrying the P301L mutation (Supporting Information Fig. S1A). The P301L mutation impairs tau binding to microtubules, thereby accelerating tangle formation and exacerbating tau pathology^17^^,^^18^. We then transfected this construct into SH-SY5Y cells and applied fluorescence-activated cell sorting to sort single cells. After cell expansion, we acquired several cell lines with tau-EGFP expression (Fig. S1B). We used Line5 (named SH-SY5Y tau(P301L)-EGFP) for the following studies. Immunofluorescence staining showed significant colocalization of GFP with total tau and various phosphorylated tau forms (pT181, pS199, and pS404) in SH-SY5Y tau(P301L)-EGFP cells (Fig. S1C).

**Figure 1:**
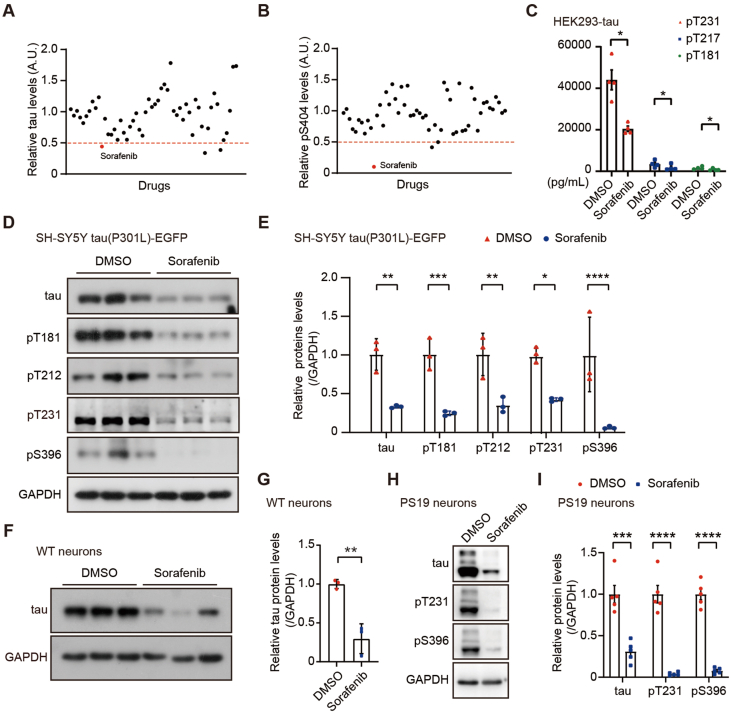
Sorafenib treatment reduces tau levels *in vitro*. (A, B) SH-SY5Y tau(P301L)-EGFP cells were treated with 54 FDA-approved drugs (10 μmol/L) or DMSO control for 24 h. Protein levels of total tau (A) and phosphorylated tau pS404 (B) were analyzed by immunoblotting, and their levels relative to those of controls were determined. (C) HEK293 cells stably expressing tau (HEK293-tau) were treated with sorafenib (10 μmol/L) or DMSO control for 24 h. Equal amounts of protein lysates were subjected to a single-molecule immunoassay to determine phosphorylated tau pT181, pT217, and pT231 levels. *n* = 4. Paired Student's *t*-test. (D, E) SH-SY5Y tau(P301L)-EGFP cells were treated with sorafenib (10 μmol/L) or DMSO control for 24 h. Equal amounts of protein lysates were analyzed by immunoblotting for total tau and phosphorylated tau, including pT181, pT212, pT231, and pS396 (D). Protein levels were quantified by densitometry for comparison (E). *n* = 3. Unpaired Student's *t*-test. (F, G) Wild-type (WT) primary neurons derived from C57BL6/J mice were treated with sorafenib (10 μmol/L) or DMSO control for 12 h. Equal amounts of protein lysates were analyzed by immunoblotting (F). Total tau levels were quantified for comparison (G). *n* = 3. Unpaired Student's *t*-test. (H, I) Primary neurons derived from PS19 mice were treated with sorafenib (10 μmol/L) or DMSO control for 12 h. Equal amounts of protein lysates were analyzed by immunoblotting (H). Protein levels of total tau and phosphorylated tau (pT231 and pS396) were quantified for comparison (I). *n* = 5. Unpaired Student's *t*-test. Data are presented as mean ± SEM; ∗*P* < 0.05; ∗∗*P* < 0.01; ∗∗∗*P* < 0.001; ∗∗∗∗*P* < 0.0001.

We subsequently treated SH-SY5Y tau(P301L)-EGFP cells with 54 FDA-approved drugs that can cross the blood-brain barrier and studied the changes in total tau and tau pS404 levels. Among these tested drugs, we found that sorafenib reduced total tau levels and had the most effect on reducing tau pS404 levels (Fig. 1A and B, Supporting Information Fig. S2A and S2B). Next, we measured the cytotoxicity of sorafenib to SH-SY5Y tau (P301L)-EGFP cells. Consistent with previous studies^19^, we found that treatment with 10 μmol/L sorafenib for up to 24 h had no significant effect on cell viability (Supporting Information Fig. S3A), and the IC_50_ of sorafenib on cell viability was 17.34 μmol/L (Fig. S3B). Therefore, we used 10 μmol/L sorafenib for subsequent cell experiments.

By using an ultrasensitive single-molecule detection assay, we found that sorafenib treatment significantly reduced tau phosphorylation at sites T181, T217, and T231 in HEK293 cells stably expressing tau (HEK293-tau) (Fig. 1C). Furthermore, we applied immunoblotting to confirm that sorafenib treatment significantly reduced the levels of total tau and various phosphorylated tau forms in SH-SY5Y tau(P301L)-EGFP cells (Fig. 1D and E), in primary neurons derived from wild type (WT) mice (Fig. 1F and G), and in primary neurons derived from PS19 mice overexpressing human tau P301S (Fig. 1H and I).

### 3.2 Sorafenib attenuates cognitive deficits and disease-related pathologies in PS19 mice

**Figure 2:**
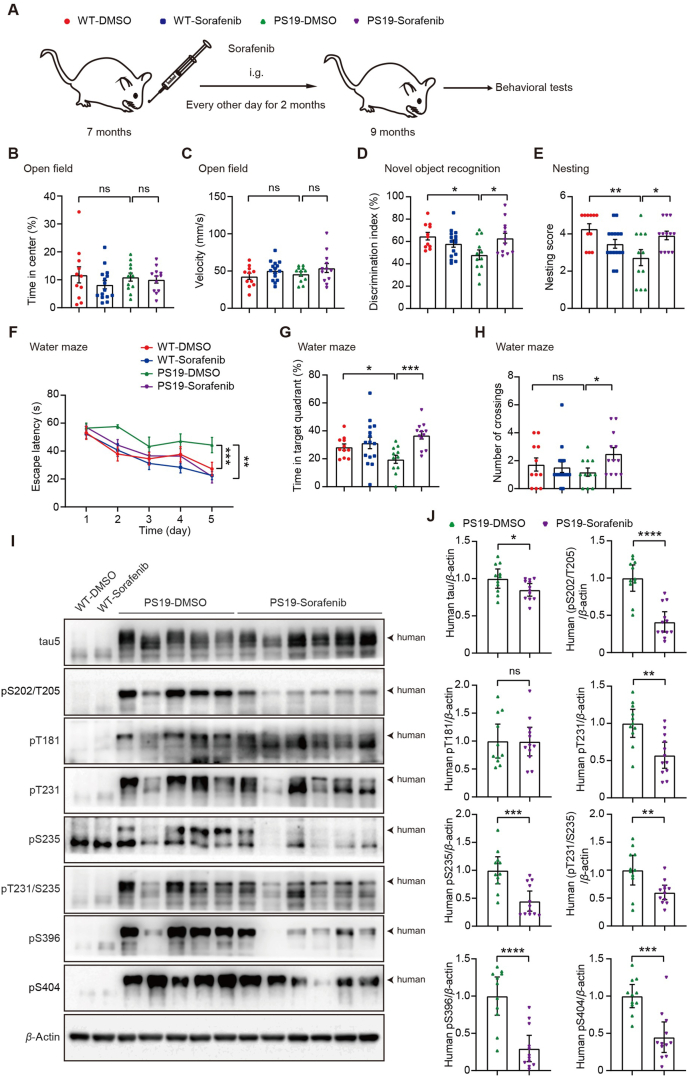
Sorafenib treatment attenuates cognitive impairments and tau pathologies in PS19 mice. (A) 7-month-old PS19 male mice and their WT littermate male controls were subjected to intragastric administration of sorafenib (10 mg/kg) or DMSO control every other day for 2 months, followed by behavioral analysis. (B, C) In the open field test, the time spent in the central region (B) and the velocity (C) were counted for comparison. One-way ANOVA with Tukey's multiple comparisons test. (D) In the novel object recognition test, the index for discriminating between the novel and the familiar object by mice was determined for comparison. One-way ANOVA with Tukey's multiple comparisons test. (E) In the nest-building test, the nesting status was scored for comparison. One-way ANOVA with Tukey's multiple comparisons test. (F) During a five-day training period of the Morris water maze test, the escape latency was determined for comparison. Two-way ANOVA with Tukey's multiple comparisons test. (G, H) During the testing period of the Morris water maze test, the time spent in the target quadrant (G) and the number of platform crossings (H) were calculated for comparison. One-way ANOVA with Tukey's multiple comparisons test. *n* = 11 mice for WT-DMSO, *n* = 15 mice for WT-Sorafenib, *n* = 11 mice for PS19-DMSO, and *n* = 12 mice for PS19-Sorafenib for all behavioral analysis. (I, J) Equal protein amounts of hippocampal tissues of treated PS19 mice were subjected to immunoblotting for the proteins indicated (I). Protein levels were quantified for comparison (J). *n* = 11 mice for PS19-DMSO, and *n* = 12 mice for PS19-Sorafenib. Unpaired Student's *t*-test. Data are presented as mean ± SEM; ∗*P* < 0.05; ∗∗*P* < 0.01; ∗∗∗*P* < 0.001; ∗∗∗∗*P* < 0.0001; ns: not significant.

Given that sorafenib can reduce tau and phosphorylated tau levels *in vitro*, we next studied whether sorafenib protects against tauopathy *in vivo*. We administered sorafenib (10 mg/kg) or DMSO vehicle control to 7-month-old WT and PS19 male mice by gavage every other day for 2 months (Fig. 2A), and subsequently evaluated their behavioral changes. We found that although the body weights of PS19 mice were consistently lighter than those of WT mice, sorafenib treatment had no significant effect on the body weights of either group (Supporting Information Fig. S4), suggesting that sorafenib treatment did not impact the overall health of these mice.

In the open-field test, there was no significant difference in the time spent in the central area between the sorafenib treatment group and the control group (Fig. 2B), indicating that sorafenib did not elicit anxiety. Mouse movement speeds were comparable among all experimental groups (Fig. 2C). In the novel object recognition test, PS19 control (PS19-DMSO) mice exhibited a decreased discrimination between novel and familiar objects compared to WT control (WT-DMSO) mice. In contrast, sorafenib-treated PS19 (PS19-Sorafenib) mice showed significantly improved discrimination index compared to PS10-DMSO mice (Fig. 2D). In the nest-building test, PS19-DMSO mice had worse nest-building scores than WT-DMSO mice; and this was reversed by sorafenib treatment (Fig. 2E). In the Morris water maze test, PS19-DMSO mice spent more time finding the hidden platform during the training phase than WT-DMSO mice, whereas PS19-Sorafenib mice spent less time than PS19-DMSO mice (Fig. 2F). In the testing stage of the Morris water maze test, PS19-DMSO mice spend less time in the target quadrant than WT-DMSO mice (Fig. 2G). Meanwhile, PS19-Sorafenib mice spent more time in the target quadrant and crossed the platform location more frequently than PS19-DMSO mice (Fig. 2G and H). Together, these results demonstrate that sorafenib treatment attenuates cognitive deficits in PS19 mice.

Moreover, we studied and found that sorafenib treatment significantly reduced the levels of total human tau and several phosphorylated human tau forms, including pS202/T205, pT231, pS235, pT231/S235, pS396, and pS404, in the hippocampus of PS19 mice, without affecting the levels of pT181 (Fig. 2I and J). Consistently, sorafenib treatment significantly reduced the levels of total human tau and all tested phosphorylated human tau forms in the cortex of PS19 mice (Supporting Information Fig. S5A and S5B). Surprisingly, although sorafenib treatment reduced total mouse tau levels in WT primary neurons (Fig. 1F), sorafenib treatment *in vivo* did not significantly affect total mouse tau levels in the hippocampus and cortex of WT mice, but reduced pT181, pS396, and pS404 of mouse tau in the hippocampus and pS404 of mouse tau in the cortex (Supporting Information Fig. S6A–S6D). Why sorafenib preferentially reduces exogenous pathogenic human tau to endogenous mouse tau *in vivo* is currently unclear. One possibility is that the effect of sorafenib on reducing total tau under physiological conditions *in vivo* may be offset by other cells, such as microglia and astrocytes. However, altered microglia and astrocytes at pathological stages may lose these offset functions, and this deserves further investigation.

Since gliosis, neuroinflammation, and oxidative stress are also pathological features in tauopathy^20^, we studied and found that sorafenib treatment significantly reduced the increased Iba1 levels in the hippocampus and cortex of PS19 mice (Fig. S5A–S5F), and the increased mRNA expression of pro-inflammatory factors including *Tnfα* and *Il1β* in the hippocampus of PS19 mice (Fig. S5G). These results indicate that sorafenib treatment reduces microgliosis and neuroinflammation in PS19 mice. However, sorafenib treatment did not reverse the increased GFAP levels (Fig. S5A–S5F) and increased mRNA expression of oxidative stress markers, including *Nfe2l2*, *Hmox1*, and *Cybb* (Fig. S5G), suggesting that sorafenib treatment did not affect astrogliosis and oxidative stress in PS19 mice. Moreover, we found that sorafenib treatment increased NeuN levels in PS19 mice (Fig. S5A–S5D), further supporting that sorafenib treatment protects against neurodegeneration.

### 3.3 Sorafenib treatment inhibits multiple kinases for tau phosphorylation

Tau protein undergoes numerous phosphorylation modifications by various kinases such as cyclin-dependent kinase 5 (CDK5), extracellular signal-regulated protein kinase (ERK), glycogen synthase kinase-3*β* (GSK3*β*), and p38^21^^,^^22^. Considering that sorafenib is a multi-kinase inhibitor^9^^,^^10^, we hypothesized that sorafenib reduces phosphorylated tau levels by modulating these kinases. We thus investigated the protein levels of these kinases and their phosphorylated forms, which affect their activities. The results showed that sorafenib treatment reduced the protein levels of CDK5, ERK1/2, and p38, and suppressed the phosphorylation of ERK1/2, p38, and GSK3*β* in SH-SY5Y tau(P301L)-EGFP cells (Supporting Information Fig. S7A and S7B). In the hippocampus of PS19 mice treated with sorafenib, the levels of CDK5 and the phosphorylation of ERK1/2, p38, and GSK3*β* were markedly decreased compared to control mice (Fig. S7C and S7D). These findings suggest that sorafenib can reduce the phosphorylation modifications of tau by inhibiting multiple kinases, including CDK5, ERK1/2, GSK3*β*, and p38.

### 3.4 Sorafenib treatment inhibits the mTOR pathway and promotes autophagy

**Figure 3:**
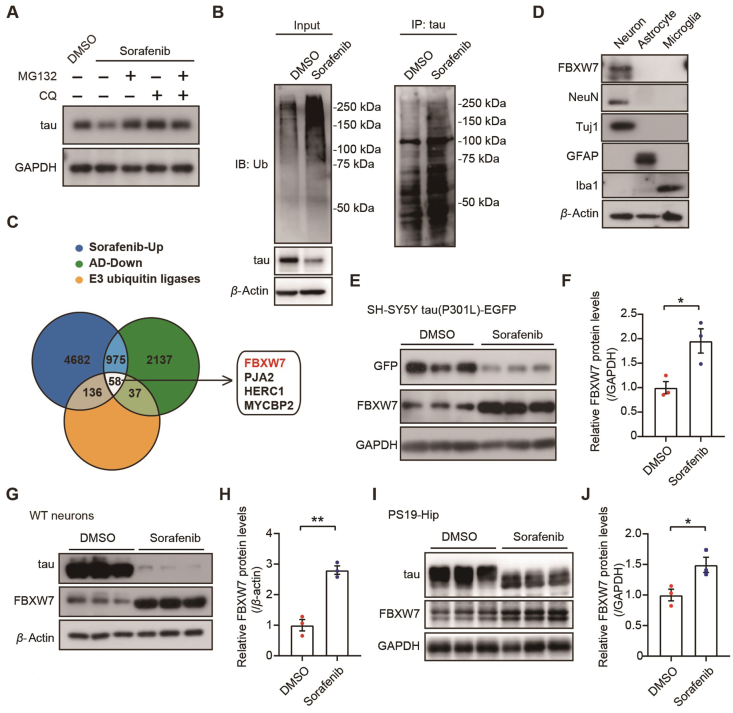
Sorafenib treatment promotes tau ubiquitination and FBXW7 levels. (A) SH-SY5Y tau(P301L)-EGFP cells were treated with sorafenib (10 μmol/L) or DMSO control for 24 h, and the proteasome inhibitor MG132 (10 μmol/L) and/or the lysosome inhibitor chloroquine (CQ, 10 μmol/L) were added in the sorafenib-treatment group 10 h before sample collection. Equal amounts of protein lysates were analyzed by immunoblotting. (B) HEK293-tau cells were treated with 10 μmol/L Sorafenib or DMSO for 24 h. Equal amounts of protein lysates were subjected to immunoprecipitation (IP) with an anti-tau antibody and then immunoblotting (IB) with an anti-ubiquitin antibody. (C) Venn diagram analysis of the intersection between known E3 ubiquitin genes and differentially expressed genes downregulated in AD (GSE36980) and upregulated upon Sorafenib treatment (GSE186280). (D) Equal amounts of protein lysates from cultured primary neurons, astrocytes, and microglia derived from C57BL6/J WT mice were immunoblotted for FBXW7. NeuN and Tuj1 are neuronal markers. GFAP is an astrocytic marker. IBA1 is a microglial marker. (E–H) SH-SY5Y tau (P301L)-EGFP cells (E, F) and WT mouse primary neurons (G, H) were treated with sorafenib or DMSO. Equal amounts of protein lysates were immunoblotted for FBXW7 (E, G). FBXW7 levels were quantified for comparison (F, H). *n* = 3. Unpaired Student's *t*-test. (I, J) PS19 mice were subjected to intragastric administration of sorafenib (10 mg/kg) or DMSO control every other day for 3 months. Protein levels of tau and FBXW7 in hippocampal lysates were analyzed by immunoblotting (I). FBXW7 levels were quantified for comparison (J). *n* = 3. Unpaired Student's *t*-test. Data are presented as mean ± SEM; ∗*P* < 0.05; ∗∗*P* < 0.01.

Previous studies have shown that sorafenib is an inhibitor of mTORC1 and can inhibit the phosphorylation of mTORC1 at S2448 and TFEB at S211, thereby inducing autophagy^23^^,^^24^. Since it has been reported that tau can be degraded through the autophagy–lysosomal pathway^25^, ^26^, ^27^ and we determined that sorafenib treatment reduced total tau levels, we speculated that sorafenib might promote tau degradation by activating autophagy. In SH-SY5Y tau(P301L)-EGFP cells treated with sorafenib, we found that the phosphorylation levels of both mTOR (*p*-mTOR) and its downstream enzyme S6 (pS6) were decreased, whereas LC3Ⅱ levels were increased (Supporting Information Fig. S8A and S8B). Furthermore, we observed a significant decrease in the phosphorylation levels of mTOR and S6 in the hippocampus of PS19 mice treated with sorafenib, though LC3Ⅱ levels were not significantly altered in these mice (Fig. S8C and S8D). Moreover, we found that the lysosomal inhibitor chloroquine (CQ) could inhibit the reduction of tau by sorafenib treatment (Fig. 3A). Together, these results support the notion that sorafenib may increase tau degradation through promoting autophagy.

### 3.5 Sorafenib treatment promotes tau ubiquitination and the expression of the E3 ubiquitin ligase FBXW7

Although it has been known that tau can be degraded through the ubiquitin-proteasome (UPS) pathway^26^^,^^28^, whether and how sorafenib regulates the UPS pathway remains unclear. Herein, we discovered that the proteasomal inhibitor MG132 could restore the reduction of tau levels upon sorafenib treatment (Fig. 3A, Supporting Information Fig. S9A and S9B), suggesting that sorafenib also reduces tau levels through the UPS pathway. We further showed that after sorafenib treatment, the total ubiquitin levels and the levels of tau ubiquitin modification in cells were significantly increased (Fig. 3B). These findings indicate that sorafenib enhances tau ubiquitination and promotes its degradation through the UPS pathway.

Since UPS-mediated protein degradation relies on the specific recognition and binding of substrates by E3 ubiquitin ligases, we screened known E3 ubiquitin ligases^29^^,^^30^ that showed decreased expression in AD^31^ but increased expression upon sorafenib treatment^32^ and identified 58 potential candidates (Fig. 3C). Acknowledging the neuron-specific expression pattern of tau, we further checked the neuronal expression pattern of these candidates. Single-cell RNA sequencing data (GSE67835) revealed that FBXW7, HERC1, PJA2, and MYCBP2 are the top four E3 ubiquitin ligases with the highest mRNA expression levels in neurons^33^. One previous study reported that FBXW7 mutations may lead to a novel neurodevelopmental disorder with varying degrees of developmental delay and intellectual disability^34^, implicating its involvement in cognitive functions. Therefore, we selected FBXW7 for further investigation here. Whether and how HERC1, PJA2, and MYCBP2 participate in tauopathy and mediate the protective effect of sorafenib requires further scrutiny.

Post-transcriptional alternative splicing of FBXW7 yields three isoforms: FBXW7*α*, FBXW7*β*, and FBXW7*γ*. Interestingly, these isoforms localize to different cellular compartments, with FBXW7*α* predominantly in the nucleus, FBXW7*β* in the cytoplasm, and FBXW7*γ* in the nucleolus^35^^,^^36^. Given that tau is predominantly localized to the neuronal cytoplasm^37^^,^^38^, we focused on FBXW7*β* in the following study and used FBXW7 to represent FBXW7*β* thereafter. We analyzed the expression pattern of FBXW7 in primary neurons, astrocytes, and microglia, and confirmed its specific expression in neurons (Fig. 3D). In addition, we showed that sorafenib treatment significantly increased the mRNA and protein levels of FBXW7 in SH-SY5Y tau(P301L)-EGFP cells (Fig. 3E and F, Supporting Information Fig. S10A), the protein levels of FBXW7 in WT primary neurons (Fig. 3G and H), and the mRNA and protein levels of FBXW7 in the hippocampus of PS19 mice (Fig. 3I and J, Fig. S10B).

### 3.6 FBXW7 levels are significantly decreased in AD and tau transgenic mice

**Figure 4:**
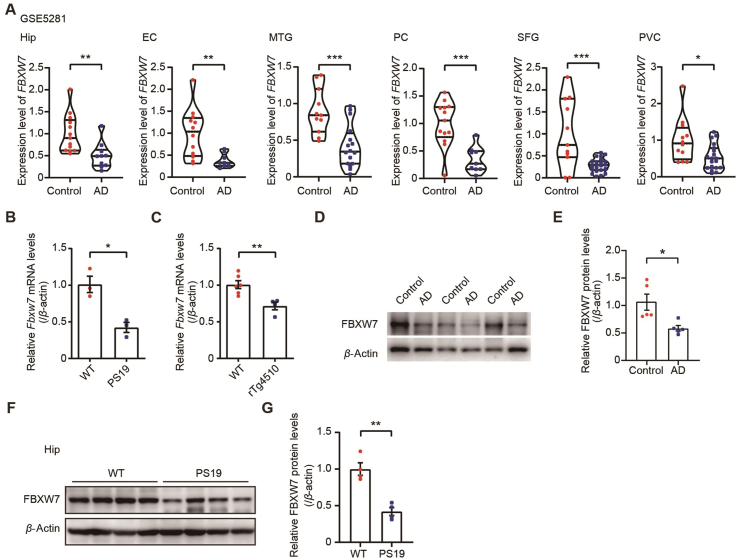
FBXW7 expression is decreased in the brains of AD patients and tauopathy model mice. (A) Comparison of *FBXW7* mRNA expression levels in various brain regions of AD and control samples based on transcriptomic data from GSE5281. Hippocampus (Hip): *n* = 13 for control and 10 for AD. Entorhinal cortex (EC): *n* = 13 for control and 9 for AD. Medial temporal gyrus (MTG): *n* = 11 for control and 16 for AD. Posterior cingulate (PC): *n* = 13 for control and 9 for AD. Superior frontal gyrus (SFG): *n* = 11 for control and 23 for AD. Primary visual cortex (PVC): *n* = 12 for control and 19 for AD. Unpaired Student's *t*-test. (B) Comparison of *Fbxw7* mRNA levels in the hippocampal tissues of 8-month-old PS19 mice and WT controls. *n* = 3 per group. Unpaired Student's *t*-test. (C) Comparison of *Fbxw7* mRNA levels in the hippocampal tissues of 8-month-old rTg4510 mice and WT controls. *n* = 6 for WT mice and *n* = 4 for rTg4510 mice. Unpaired Student's *t*-test. (D, E) Equal protein amounts of postmortem cortical brain tissues of AD patients and controls were immunoblotted for FBXW7 (D), whose levels were quantified for comparison (E). *n* = 6. Unpaired Student's *t*-test. (F, G) Equal protein amounts of hippocampal tissues of 12-month-old PS19 mice and WT controls were immunoblotted for FBXW7 (F), whose levels were quantified for comparison (G). *n* = 4. Unpaired Student's *t*-test. Data are presented as mean ± SEM; ∗*P* < 0.05; ∗∗*P* < 0.01; ∗∗∗*P* < 0.001.

To further investigate the correlation between FBXW7 and tauopathy, we analyzed the GSE5281 AD transcriptomic dataset^39^, ^40^, ^41^. We found that the mRNA levels of *FBXW7* were significantly decreased in six brain regions, including hippocampus (Hip), entorhinal cortex (EC), medial temporal gyrus (MTG), posterior cingulate cortex (PC), superior frontal gyrus (SFG), and visual cortex (VCX) in AD patients compared to controls (Fig. 4A).

Subsequently, we examined the mRNA levels of *Fbxw7* in the hippocampus of different tau transgenic mice, including PS19 and rTg4510 (overexpressing human tau P301L). qRT-PCR analyses revealed a significant decrease in *Fbxw7* mRNA levels in PS19 and rTg4510 mice compared to their respective controls (Fig. 4B and C, Fig. S10B). FBXW7 protein levels were consistently dramatically decreased in the cortical tissues of AD patients (Fig. 4D and E) and the hippocampus of PS19 mice compared to their respective controls (Fig. 4F and G). The reduction in FBXW7 in the brains of AD patients and the tauopathy mouse models implicates an involvement of FBXW7 in the pathogenesis of tauopathy.

### 3.7 FBXW7 regulates tau degradation through the UPS pathway

**Figure 5:**
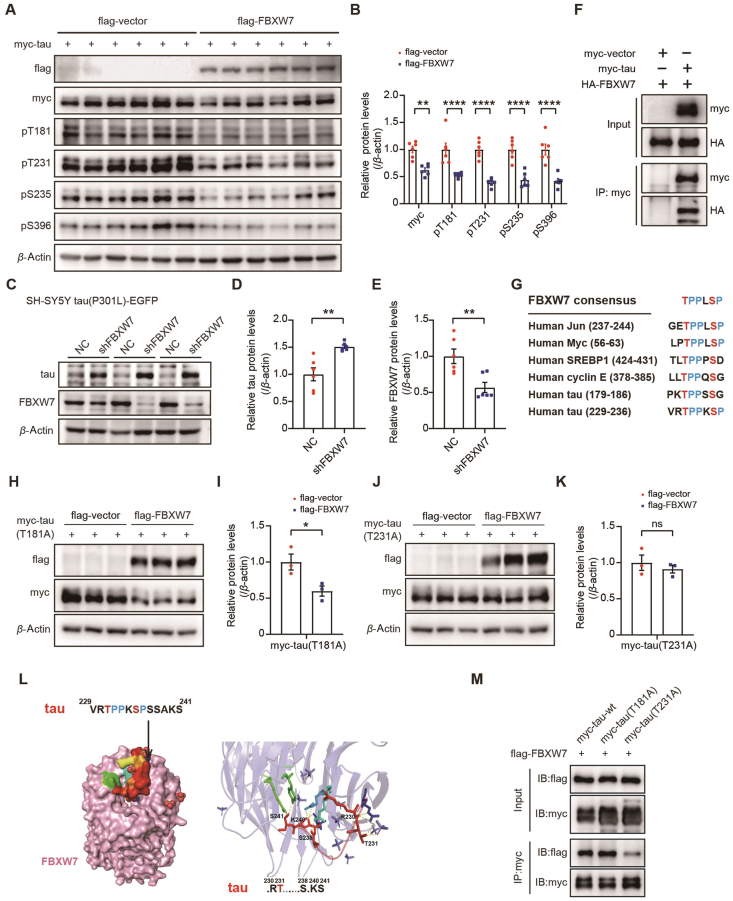
FBXW7 overexpression reduces tau levels. (A, B) HEK293T cells were transfected with myc-tau for 12 h, and then split into two groups for transfection with either a flag vector control or flag-FBXW7, respectively. Total tau and phosphorylated tau protein levels were immunoblotted (A) and quantified for comparison (B). *n* = 6. Unpaired Student *t*-test. (C–E) SH-SY5Y tau(P301L)-EGFP cells were infected with lentiviruses expressing FBXW7 shRNA (shFBXW7) or negative control (NC) for 72 h. Equal amounts of protein lysates were immunoblotted for the proteins indicated (C). Total tau (D) and FBXW7 (E) levels were quantified for comparison. *n* = 3. Unpaired Student's *t*-test. (F) HEK293T cells were transfected with HA-FBXW7 for 12 h, and then split into two groups for transfection with either a myc vector control or myc-tau, respectively. Equal amounts of protein lysates were subjected to immunoprecipitation (IP) with an anti-myc antibody and then immunoblotting with anti-myc and anti-HA antibodies. (G) Sequence comparison of the FBXW7-binding motif in known FBXW7 substrates and in tau. (H–K) Flag-FBXW7 was co-transfected with myc-tau(T181A) mutant (H, I) or myc-tau(T231A) mutant (J, K) into HEK293T cells. Equal amounts of protein lysates were immunoblotted for the proteins indicated (H, J). Myc-tau(T181A) mutant (I) and myc-tau(T231A) mutant (K) levels were quantified for comparison. *n* = 3. Unpaired Student's *t*-test. (L) Molecular docking predicts a binding of FBXW7 to the tau 229-241 motif. (M) HEK293T cells were transfected with Flag-FBXW7 for 12 h, and then split into three groups for transfection with WT myc-tau, myc-tau(T181A), and myc-tau(T231A), respectively. Equal amounts of protein lysates were subjected to IP with an anti-myc antibody and then immunoblotting (IB) with anti-flag and anti-myc antibodies. Data are presented as mean ± SEM; ∗*P* < 0.05; ∗∗*P* < 0.01; ∗∗∗∗*P* < 0.0001; ns: not significant.

Since FBXW7 is an E3 ubiquitin ligase, we studied whether tau could be a substrate of FBXW7. In HEK293T cells overexpressing myc-tau, we found that overexpression of FBXW7 significantly reduced protein levels of total tau and multiple phosphorylated tau forms (Fig. 5A and B). In contrast, *FBXW7* knockdown in SH-SY5Y tau(P301L)-EGFP cells significantly increased tau protein levels (Fig. 5C–E).

In HEK293T cells expressing myc-tau and HA-FBXW7, we found that an anti-myc antibody immunoprecipitated both myc-tau and HA-FBXW7, indicating the interaction between FBXW7 and tau (Fig. 5F). In addition, an anti-tau antibody immunoprecipitated both endogenous tau and endogenous FBXW7 in mouse hippocampal samples (Supporting Information Fig. S11A), confirming the tau-FBXW7 interaction. Immunofluorescence study also revealed a significant co-localization of tau and FBXW7 in primary neurons of PS19 mice (Fig. S11B). Furthermore, FBXW7 overexpression promoted tau ubiquitination (Fig. S11C). Together, these results suggest that FBXW7 interacts with tau and regulates its degradation through the UPS pathway.

Moreover, we found that sorafenib treatment elevated the binding affinity between FBXW7 and tau (Fig. S11D). Meanwhile, sorafenib-induced tau reduction was alleviated upon *FBXW7* knockdown (Fig. S11E and S11F). These results indicate that sorafenib treatment reduces tau levels also through promoting FBXW7-mediated tau degradation.

It is suggested that FBXW7 promotes the ubiquitination and degradation of substrates through substrate-dependent phosphorylation, and most of its substrates, such as Jun, Myc, SREBP1, and cyclin E, contain conserved amino acid sequences for phosphorylation, also known as the CDC4 phospho-degron (CPD)^42^. The phosphorylation of S/T residues within the CPD sequence of the substrate plays a crucial role in the binding of FBXW7 to the substrate^43^. Through sequence alignment analysis of the conserved sequences of tau (2N4R) and known substrates of FBXW7, we identified two potential FBXW7-binding sequences of tau, located at the T181 site (^179^PKTPPSSG^186^) and the T231 site (^229^VRTPPKSP^236^) (Fig. 5G). To verify whether phosphorylation at the T181 and/or T231 site affects the degradation of tau by FBXW7, we constructed phosphorylation-inactivating mutation plasmids: myc-tau(T181A) and myc-tau(T231A). When myc-tau(T181A) was expressed in HEK293T cells, additional expression of FBXW7 significantly reduced myc-tau(T181A) levels (Fig. 5H and I). However, FBXW7 expression did not affect myc-tau(T231A) levels (Fig. 5J and K), implicating that T231 phosphorylation is crucial for FBXW7-mediated tau degradation. Molecular docking also predicted a binding between FBXW7 and a tau peptide segment (positions 229–241) that includes the T231 site (Fig. 5L). Moreover, we found that the tau(T231A) mutant had decreased binding ability to FBXW7 compared to WT tau and the tau(T181A) mutant using co-immunoprecipitation assays (Fig. 5M). These results suggest that the CPD sequence of tau is located at the T231 site (^229^VRTPPKSP^236^) and that FBXW7 relies on T231 phosphorylation for mediating tau degradation.

### 3.8 Overexpression of FBXW7 ameliorates cognitive impairments and tau pathologies in PS19 mice

**Figure 6:**
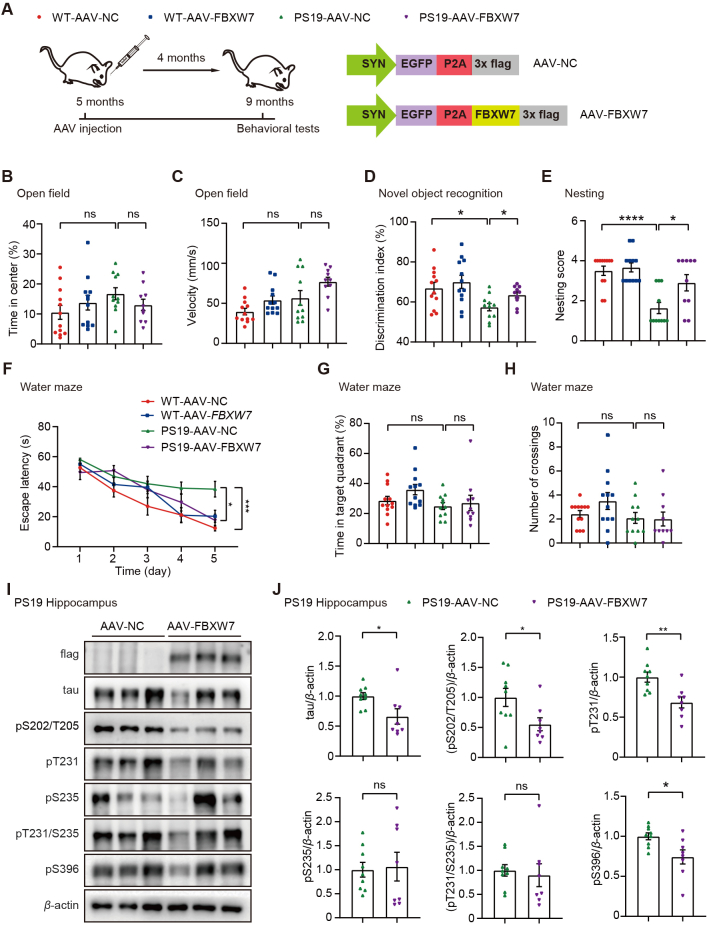
FBXW7 overexpression attenuates cognitive deficits and tau pathologies in PS19 mice. (A) 5-month-old male mice, including PS19 and WT littermate controls, were subjected to hippocampal CA1 region injection of AAV-FBXW7 or NC control. After 4 months, the mice were analyzed for their behaviors. (B, C) In the open-field test, time spent in the central area (B) and the velocity (C) were counted for comparison. One-way ANOVA with Tukey's multiple comparisons test. (D) In the novel object recognition test, the index for discriminating between the novel and the familiar object by mice was determined for comparison. One-way ANOVA with Tukey's multiple comparisons test. (E) In the nest-building test, the nesting status was scored for comparison. One-way ANOVA with Tukey's multiple comparisons test. (F) During a five-day training period of the Morris water maze test, the escape latency was determined for comparison. Two-way ANOVA with Tukey's multiple comparisons test. (G, H) During the testing period of the Morris water maze test, the time spent in the target quadrant (G) and the number of platform crossings (H) were counted for comparison. One-way ANOVA with Tukey's multiple comparisons test. *n* = 12 mice for WT-AAV-NC, *n* = 12 mice for WT-AAV-FBXW7, *n* = 11 mice for PS19-AAV-NC, and *n* = 10 mice for PS19-S AAV-FBXW7 for all behavioral analysis. (I, J) Equal protein amounts of hippocampal tissues of treated PS19 mice were subjected to immunoblotting for the proteins indicated (I). Protein levels were quantified for comparison (J). *n* = 9 mice for PS19-AAV-NC, and *n* = 8 mice for PS19-AAV-FBXW7. Unpaired Student's *t*-test. Data are presented as mean ± SEM; ∗*P* < 0.05; ∗∗*P* < 0.01; ∗∗∗∗*P* < 0.0001; ns: not significant.

Given the diminished FBXW7 expression in PS19 mice and its role in regulating tau degradation, we investigated the potential of elevating FBXW7 *in vivo* to mitigate tau pathology. We administered adeno-associated viruses (AAVs) overexpressing *FBXW7* (AAV-FBXW7) or control (AAV-NC) into 5-month-old wild-type and PS19 male mice *via* stereotactic brain injection. After four months, we studied animal behaviors (Fig. 6A).

In the open field test, we found that FBXW7 overexpression did not affect locomotor activity and the time spent in the center for these mice (Fig. 6B and C). In the novel object recognition test, FBXW7 overexpression significantly attenuated the decreased discrimination index in PS19 mice (Fig. 6D). FBXW7 overexpression also rescued the impaired nest-building ability of PS19 mice (Fig. 6E). In the training stage of the Morris water maze test, we also found that PS19 control mice spent more time finding the hidden platform than WT controls. In contrast, overexpression of FBXW7 in PS19 shortened their escape latency (Fig. 6F). However, in this batch of mice, PS19 mice showed no altered time spent in the target quadrant and numbers of platform crossings compared to WT controls during the testing phase of the Morris water maze test, and FBXW7 had no effects on these parameters (Fig. 6G and H). Furthermore, we found that in the three-chamber social interaction test, PS19 mice showed no differences in their social interaction behaviors (Supporting Information Fig. S12A and S12B) but exhibited social memory deficits compared to WT control mice, which were ameliorated by FBXW7 overexpression (Fig. S12C).

We also studied the hippocampal tissues of these treated mice. The results showed that FBXW7 overexpression significantly reduced the levels of total tau and several phosphorylated tau, including pS202/T205, pT231, and pS396 *in vivo*. However, the levels of pS235 and pT231/S235 remained unchanged (Fig. 6I and J).

## 4 Discussion

Deposition of abnormal tau protein, especially its hyperphosphorylation forms, is not only a representative hallmark of various tauopathies, including AD, but also a crucial etiology of these devastating disorders. Therefore, reducing tau and/or phosphorylated tau levels may be an effective strategy for treating tauopathies^2^^,^^5^. Multiple strategies for this purpose have been under development, such as using antisense oligomers to reduce tau expression, kinase inhibitors to reduce tau hyperphosphorylation, and antibodies to eliminate tau and phosphorylated tau^2^^,^^44^. However, these efforts have not yielded significant efficacy in human clinical trials for successful market launch^2^^,^^5^. New drug development is notoriously lengthy and costly, averaging 10–15 years and billions of dollars^45^^,^^46^. The blood–brain barrier increases the complexity of drug development for central nervous system disorders, such as tauopathies^47^. Therefore, repurposing existing drugs, which have demonstrated clinical safety, may decrease research and development costs and shorten the development timeline.

In the present study, we employed a drug repurposing strategy to evaluate 54 FDA-approved drugs that can pass the blood–brain barrier for their ability to reduce tau and phosphorylated tau levels. In cells expressing tau, we identified sorafenib as the most effective drug in reducing both tau and phosphorylated tau levels.

Sorafenib is a small-molecule tyrosine kinase inhibitor used for cancer treatment^7^^,^^8^. Some studies have found that sorafenib treatment also reduces NF-*κ*B and its downstream targets COX-2 and iNOS and improves working memory in aged APPswe mice, an AD model with A*β* pathologies^12^. Additionally, sorafenib reduces both microgliosis and astrogliosis in mice treated with LPS and astrogliosis in 5xFAD mice, another AD model with A*β* pathologies^11^. However, sorafenib treatment did not reduce A*β* levels in APPswe mice^12^, implicating that the protective effect of sorafenib is not through attenuating A*β* toxicity. Another study reported that sorafenib treatment attenuated neurodegeneration in *Caenorhabditis elegans* and *Drosophila* models of Parkinson's disease (PD) by inhibiting LRRK2 kinase activity^48^. However, it is unknown whether sorafenib can attenuate *α*-synuclein aggregates, a crucial pathological feature of PD. These findings suggest a potential role for sorafenib in the treatment of neurodegenerative diseases. Herein, we further found that oral administration of sorafenib significantly improved the cognitive function of PS19 mice, especially their learning and memory abilities. Moreover, we showed that sorafenib treatment attenuated tau pathologies by reducing total tau and multiple phosphorylated tau forms. Additionally, sorafenib treatment alleviated microgliosis and neuroinflammation, though it had no apparent effects on astrogliosis and oxidative stress. Together, these results indicate that sorafenib may also have therapeutic value for tauopathies.

There are at least 85 phosphorylation modification sites on tau, and some phosphorylated tau forms (*e*.*g*., p-tau181, p-tau217, and p-tau231) are significantly increased in the early stages of AD, implicating their contribution to disease progression and providing potential biomarkers for early diagnosis of AD^49^^,^^50^. Tau can be phosphorylated by different kinases such as GSK-3*β*, CDK5, p38, and ERK1/2^21^^,^^22^. In the present study, we found that sorafenib treatment dramatically reduced levels of these kinases and/or their active phosphorylated forms both *in vitro* and *in vivo*, suggesting that inhibition of these kinases is one of the mechanisms responsible for the reduction of tau phosphorylation upon sorafenib treatment.

Tau protein degradation is mediated by the autophagy–lysosome and the UPS pathways, both of which have been shown to be impaired during tauopathy^25^^,^^26^^,^^28^^,^^51^. Therefore, enhancing autophagy and UPS function is considered a promising therapeutic strategy for tauopathies. Some previous studies have found that sorafenib can inhibit the mTOR signaling pathway and induce autophagy^23^^,^^24^. Herein, we also found that sorafenib treatment reduced total tau levels and inhibited mTOR signaling *in vitro* and *in vivo*, suggesting that sorafenib treatment promotes tau degradation by enhancing the autophagy–lysosomal pathway.

More importantly, we found that sorafenib also promoted the ubiquitination and proteasome-mediated degradation of tau. The regulation of sorafenib on the UPS pathway was previously unknown. Herein, we demonstrated that sorafenib treatment promoted the expression of FBXW7, an E3 ubiquitin ligase crucial for the UPS pathway. One study found that the ERK kinase could phosphorylate the T205 site of FBWX7 to facilitate its degradation^52^. Since sorafenib is also an inhibitor of the RAS–MAPK–ERK pathway^53^, and we observed that sorafenib treatment inhibited ERK1/2, sorafenib treatment may also increase FBXW7 by inhibiting the ERK signaling.

FBXW7 is a component of the SCF (Skp1–Cullin–F-box) E3 ubiquitin ligase complex and can regulate cell proliferation, apoptosis, cell cycle, and differentiation in various human cancers by degrading proto-oncogenes^35^. *FBXW7* mutations have been linked to impaired ubiquitination and a novel neurodevelopmental syndrome that exhibits developmental delay and intellectual disability^34^. Interestingly, one study reported that FBXW7 could interact with presenilin 1 (PS1), a crucial component of the *γ*-secretase that cleaves the *β*-amyloid precursor protein (APP) for A*β* generation, and enhance PS1 ubiquitination^54^. Herein, we found that FBXW7 expression was significantly decreased in various brain regions of AD patients and the hippocampus of PS19 mice. Moreover, we showed that overexpression of FBXW7 in the hippocampus attenuated cognitive deficits and tau pathologies in PS19 mice. These findings suggest that decreased FBXW7 expression contributes to the progression of AD and other tauopathies.

The phosphorylation of serine (S) or threonine (T) residues within the CDC4 phospho-degron (CPD) sequences of substrates is critical for FBXW7 binding^42^^,^^43^^,^^55^^,^^56^. We found that the probable tau CPD sequence is the ^229^VRTPPKSP^236^ motif, within which T231 phosphorylation is crucial for FBXW7 binding. Phosphorylation of tau at T231 occurs as an early event in AD neuronal pathology and precedes phosphorylation at pS202/pT205 and A*β* pathology^57^^,^^58^. High levels of tau pT231 are strongly associated with cognitive deterioration in those with mild cognitive impairment (MCI)^59^. These findings suggest that tau pT231 may become a biomarker for early AD detection, a prognostic indicator for AD progression, and a target for disease intervention. Moreover, they imply that FBXW7 expression is altered in early AD stages, so tau pT231 is less targeted for degradation by the FBXW7-mediated UPS pathway. Intriguingly, the ^230^RTPPKSP^236^ motif of tau was also reported to be a conserved binding site for the phosphatase PP2A. T231 phosphorylation could attenuate the affinity of tau for PP2A, consequently impairing the ability of PP2A to dephosphorylate other critical tau phosphorylation sites, which exacerbates the progression of tauopathies^60^^,^^61^. Therefore, FBXW7 reduction may also promote disease progression through this mechanism, and this deserves further scrutiny.

This study has several limitations. Although our current study, as well as others showed that sorafenib treatment markedly attenuated impaired cognitive skills in mice with tau pathology, with A*β* pathology^12^, and with suarachnoid hemorrhage^62^, some other studies found that C57BL/6 mice treated with 30 mg/kg sorafenib for three consecutive weeks and BALB/c mice treated with 60 mg/kg sorafenib for four consecutive weeks exhibited cognitive impairments^19^^,^^63^. In human cancer patients treated with sorafenib, the results are also controversial: a cross-sectional study reported that metastatic renal cell cancer patients treated with sunitinib or sorafenib for at least 8 weeks had worse cognitive functioning than those not treated with sunitinib or sorafenib^64^. In another study investigating sorafenib treatment of advanced hepatocarcinoma in cirrhotic patients with preserved liver function, the researchers also found that sorafenib might induce metabolic encephalopathy, leading to cognitive impairment^65^. However, in consecutive patients with cytokine-refractory kidney cancer treated with sorafenib or sunitinib, although their cognitive function and global quality of health deteriorated at Week 4 since drug treatment, such deteriorations started to reverse at Week 6. After 16 weeks of therapy, their cognitive function and global quality of health were the same as before the treatment, suggesting that sorafenib treatment does not adversely affect patient cognition during long-term treatment^66^. These discrepancies about the effects of sorafenib on cognitive functions may stem from differences in study design, patient populations, and/or assessment methodologies. In addition, these differences may be attributed to variations in the dosage and duration of treatment with sorafenib. Moreover, sorafenib has multiple functions, which, on one hand, may synergistically reduce tau and phosphorylated tau as observed here, but on the other hand, may target other proteins and pathways important for physiological functions, thereby causing adverse effects. Further elucidation of its target specificity and a thorough evaluation of its treatment settings and therapeutic window, such as dosage, frequency, treatment length, etc., are crucial for determining the clinical use of sorafenib in treating cognitive impairment.

## 5 Conclusions

In the present study, we identified the FDA-approved drug sorafenib as a potential drug for treating tauopathies. Our findings suggest that sorafenib attenuates tau pathologies through multiple pathways, including inhibiting tau kinases, elevating the autophagy–lysosomal pathway, and promoting the FBXW7-mediated UPS pathway. Moreover, we revealed that FBXW7 expression decreases in tauopathies, and restoring its levels may provide an alternative strategy for disease intervention.

## Author contributions

Yunqiang Zhou and Yunwu Zhang designed the research. Yunqiang Zhou, Yong Wang, and Huiying Yang performed most of the cellular and molecular experiments. Yunqiang Zhou and Yong Wang carried out animal experiments. Huiying Yang, Chi Zhang and Kun Li helped on plasmid construction. Jian Meng, Lingliang Zhang and Ling-ling Huang helped with data interpretation. Xian Zhang and Hong Luo provided technical support. Yunqiang Zhou and Yunwu Zhang drafted the manuscript. Yunwu Zhang supervised the research. All authors reviewed and proved the final manuscript.

## Conflicts of interest

The authors declare no conflicts of interest.

## Appendix A Supplementary data

The following is the Supporting Information to this article:
