# Supplementary material for: Sorafenib promotes the E3 ubiquitin ligase FBXW7 to increase tau degradation and ameliorate tauopathies
Source: Acta Pharm Sin B. 2025 Sep 17;15(11):5817–31. doi: 10.1016/j.apsb.2025.09.024 (PMC12648003; doi:10.1016/j.apsb.2025.09.024)
Supplement: Multimedia component 1 [file mmc1.pdf]

Supporting Information for

Original article

Sorafenib promotes the E3 ubiquitin ligase FBXW7 to increase tau degradation and ameliorate tauopathies

Yunqiang Zhou[a,†], Yong Wang[a,†], Huiying Yang[b,†], Chi Zhang[a], Jian Meng[a], Lingliang Zhang[a], Kun Li[a], Ling-ling Huang[a], Xian Zhang[a], Hong Luo[a], and Yunwu Zhang[a,b,*]

[a]*Fujian Provincial Key Laboratory of Neurodegenerative Disease and Aging Research, Institute of Neuroscience, School of Medicine, Xiamen University, Xiamen 361102, China*

[b]*Xiamen Key Laboratory of Brain Center, The First Affiliated Hospital of Xiamen University, School of Medicine, Xiamen University, Xiamen 361102, China*

Received 21 January 2025; received in revised form 14 May 2025; accepted 17 June 2025

*Corresponding author.

E-mail address: yunzhang@xmu.edu.cn (Yunwu Zhang).

[†]These authors made equal contributions to this work.

Running title: FBXW7 mediates sorafenib's protective effect in tauopathy

1. Supporting table

Table S1 Subject information.

No.	Brain bank code	Primary neuropathologic diagnosis	Age of death	Gender	Sample	Brain bank
1	2019CBB024	Control	75	Male	Human brain cortical tissue lysates	National Health and Disease Human Brain Tissue Resource Center
2	2018CBB009	Control	69	Female		
3	2019CBB036	Control	92	Male		
4	2020CBB003	Control	87	Male		
5	2018CBB006	Control	84	Female		
6	BB003	AD	88	Female		Neurodegenerative Disorder Research Center
7	BB023	AD	79	Male		
8	BB024	AD		Male		
9	BB034	AD	75	Male		
10	BB043	AD	83	Female		

2. Supporting figures

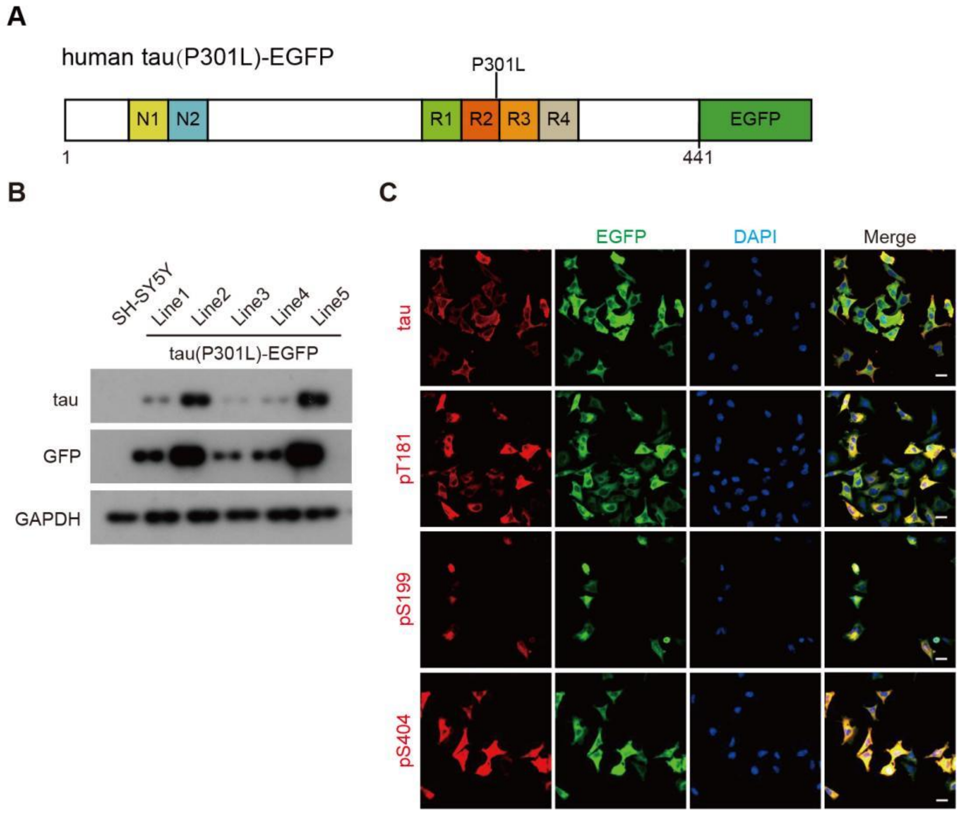

Figure S1 Construction of SH-SY5Y cell lines stably overexpressing tau(P301L)-EGFP. (A) Schematic diagram of tau(P301L)-EGFP. Full-length human tau (1–441) with the P301L mutation is fused with EGFP on the carboxyl-terminus. (B) Immunoblotting analysis of the proteins indicated in SH-SY5Y tau(P301L)-EGFP monoclonal cells. (C) Immunofluorescence of total tau (in red) and phosphorylated tau (pT181, pS199, and pS404, in red) expression in SH-SY5Y tau(P301L)-EGFP cells (Line5). The nuclei were counterstained with DAPI (in blue). EGFP signal is in green. Scale bars: 20 μm.

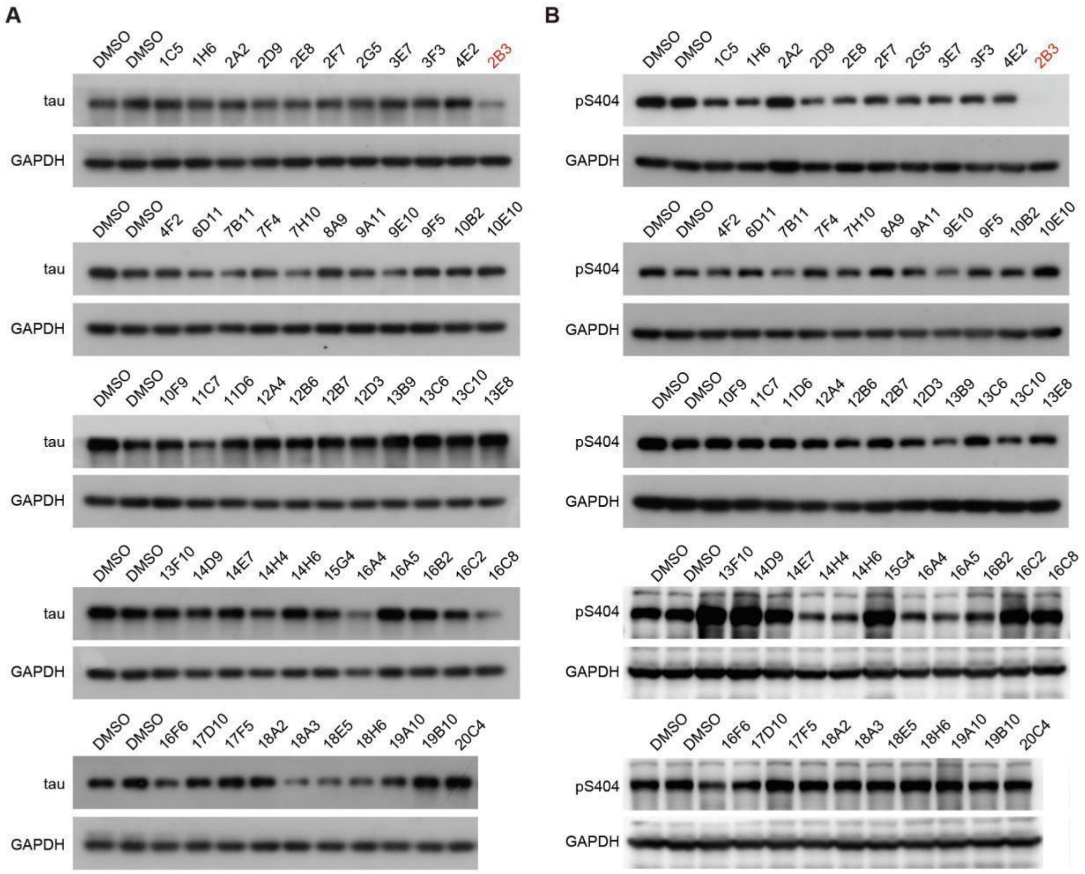

Figure S2 Screening of 54 FDA-approved drugs in the SH-SY5Y tau (P301L)-EGFP cell line. (A, B) SH-SY5Y tau(P301L)-EGFP cells were treated with 54 FDA-approved drugs (10 μmol/L) or an equal volume of the solvent control (DMSO) for 24 h, and the protein levels of total tau (A) and phosphorylated tau pS404 (B) were analyzed by immunoblotting. Sorafenib is 2B3 indicated here.

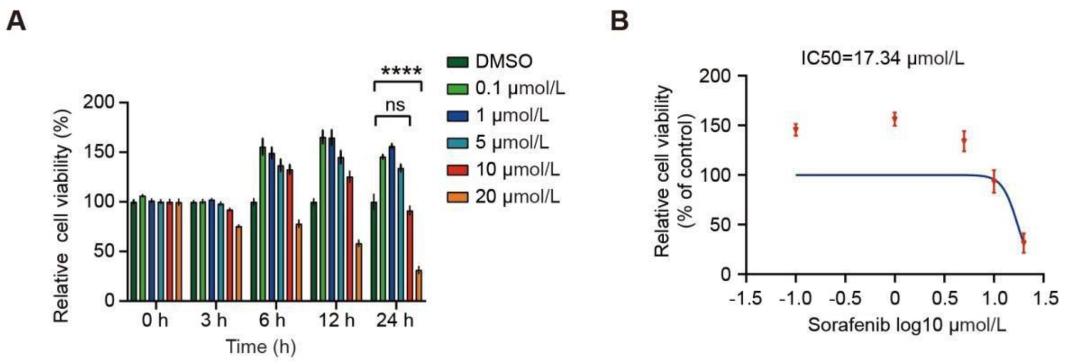

Figure S3 Cytotoxicity testing of sorafenib in SH-SY5Y tau (P301L)-EGFP cells. (A) SH-SY5Y tau(P301L)-EGFP cells were treated with different concentrations of sorafenib (0.1, 1, 5, 10, and 20 μmol/L) or vehicle control (DMSO) for different periods (0, 3, 6, 12, and 24 h). Cell viability was analyzed using the CCK-8 assay for comparison. n = 8. Two-way ANOVA with Tukey's multiple comparisons test. (B) The half-inhibitory concentration (IC_{50}) of sorafenib treatment for 24 h in (A) was determined. Data are presented as mean ± SEM; ****$P < 0.0001$; ns: not significant.

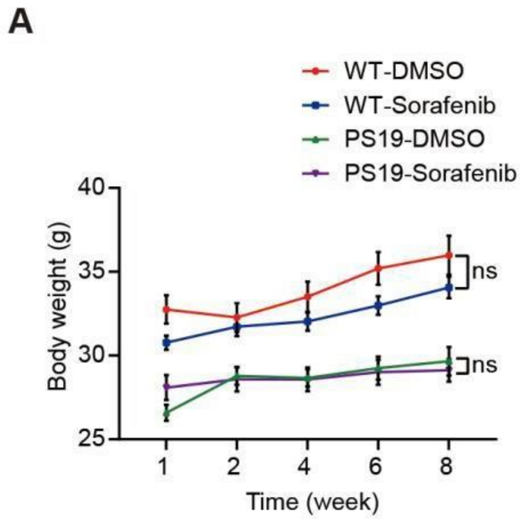

Figure S4 Sorafenib treatment does not affect mouse body weight. The body weights of mice were checked during the sorafenib treatment procedure for comparison. n =15 mice for WT-DMSO, n = 15 mice for WT-Sorafenib, n = 14 mice for PS19-DMSO, and n = 15 mice for PS19-Sorafenib. Two-way ANOVA with Tukey's multiple comparisons test. Data are presented as mean ± SEM. ns: not significant.

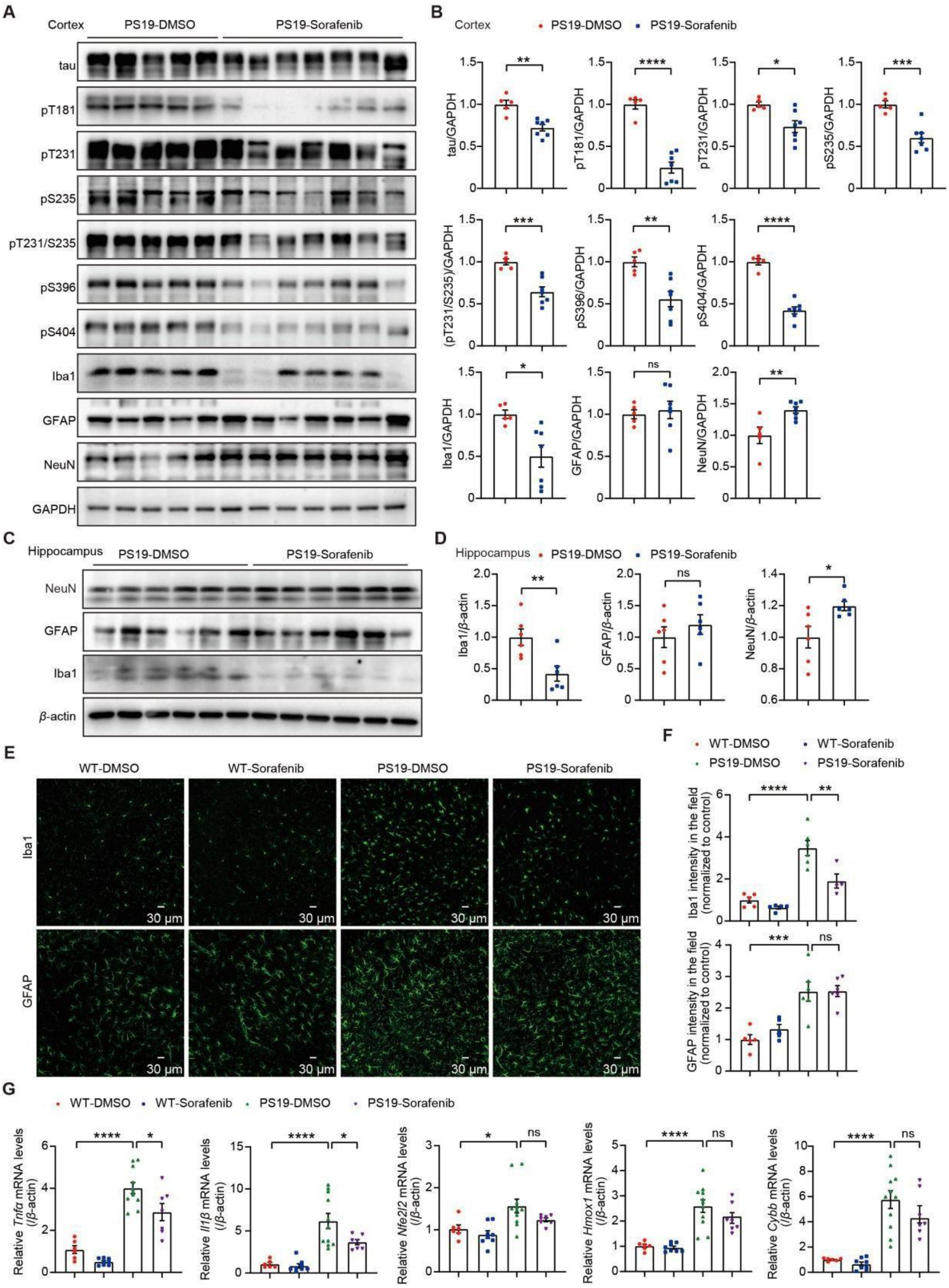

Figure S5 Sorafenib treatment attenuates tau pathologies and neuroinflammation in PS19 mice. (A, B) Equal protein amounts of cortical (Cor) tissues from sorafenib-treated PS19 mice and controls were immunoblotted for the proteins indicated (A), followed by quantitative comparisons (B). n = 5 mice for PS19-DMSO, and n = 7 mice for PS19-Sorafenib. Unpaired Student's t-test. (C, D) Equal protein amounts of hippocampal (Hip) tissues from sorafenib-treated PS19 mice and controls were immunoblotted for proteins

indicated (C), followed by quantitative comparisons (D). *n* = 6 per group. Unpaired Student's *t*-test. (E, F) Representative images showing Iba1 immunostaining indicative of microglia (in green) and GFAP immunostaining indicative of astrocytes (in green) in the hippocampal regions of WT and PS19 mice treated with or without sorafenib (E), and comparisons of Iba1 and GFAP immunointensities (F). Scale bars: 30 μm. For $Iba1^+$ microglia comparisons, *n* = 5 mice for WT-DMSO, *n* = 5 mice for WT-Sorafenib, *n* = 6 mice for PS19-DMSO, and *n* = 4 mice for PS19-Sorafenib. For $GFAP^+$ astrocyte comparisons, *n* = 5 mice for WT-DMSO, *n* = 5 mice for WT-Sorafenib, *n* = 6 mice for PS19-DMSO, and *n* = 6 mice for PS19-Sorafenib. One-way ANOVA with Tukey's multiple comparisons test. (G) The mRNA levels of indicated pro-inflammatory factors and oxidative stress markers in hippocampal tissues of sorafenib-treated WT and PS19 mice and controls were analyzed by qRT-PCR for comparison. *n* = 6 mice for WT-DMSO, *n* = 8 mice for WT- Sorafenib, *n* = 11 mice for PS19-DMSO, and *n* = 7 mice for PS19-Sorafenib. One-way ANOVA with Tukey's multiple comparisons test. Data are presented as mean ± SEM; **P* < 0.05; ***P* < 0.01; ****P* < 0.001; *****P* < 0.0001; ns: not significant.

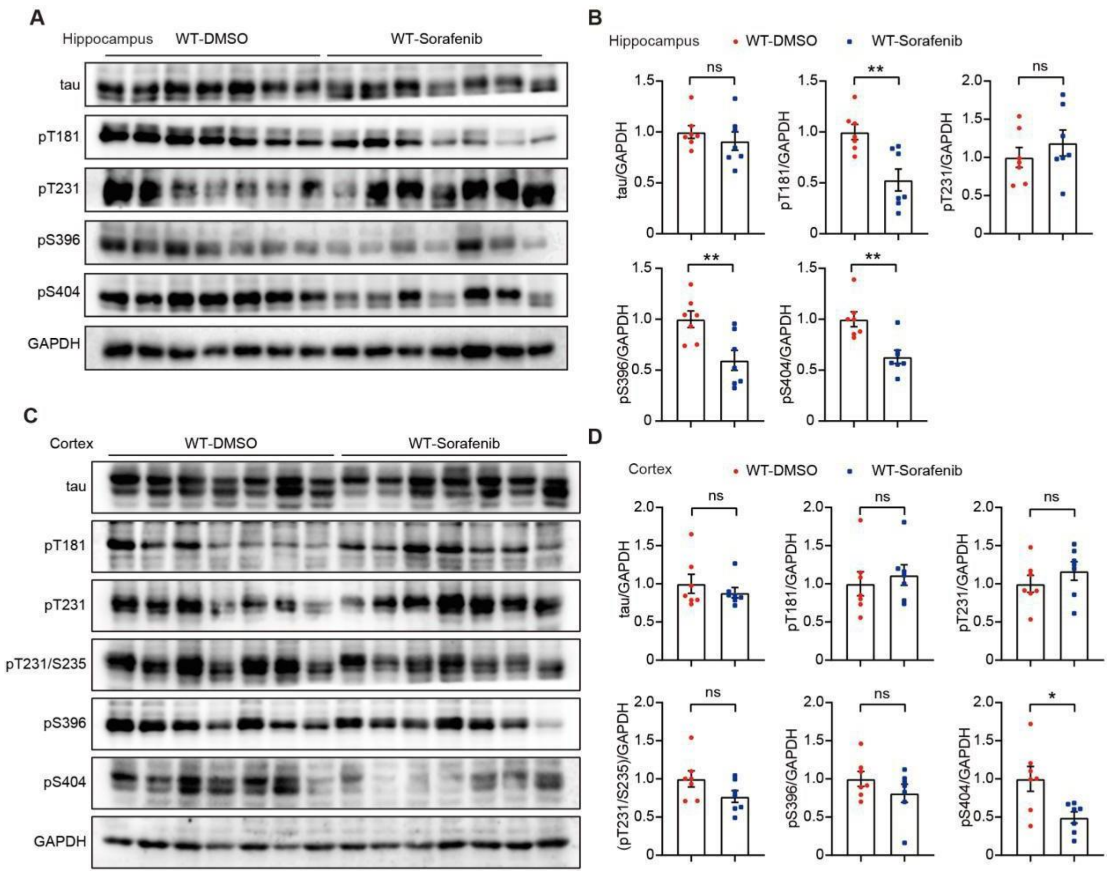

Figure S6 Sorafenib treatment does not affect total tau levels in WT mice. (A–D) Equal protein amounts of hippocampal (Hip, A, B) and cortical (Cor, C, D) tissues of WT mice treated with or without sorafenib were analyzed by immunoblotting for total and various tau phosphorylation forms (A, C), followed by quantitative comparisons (B, D). $n = 7$ per group. Unpaired Student's t-test. Data are presented as mean ± SEM; $*P < 0.05$; $**P < 0.01$; ns: not significant.

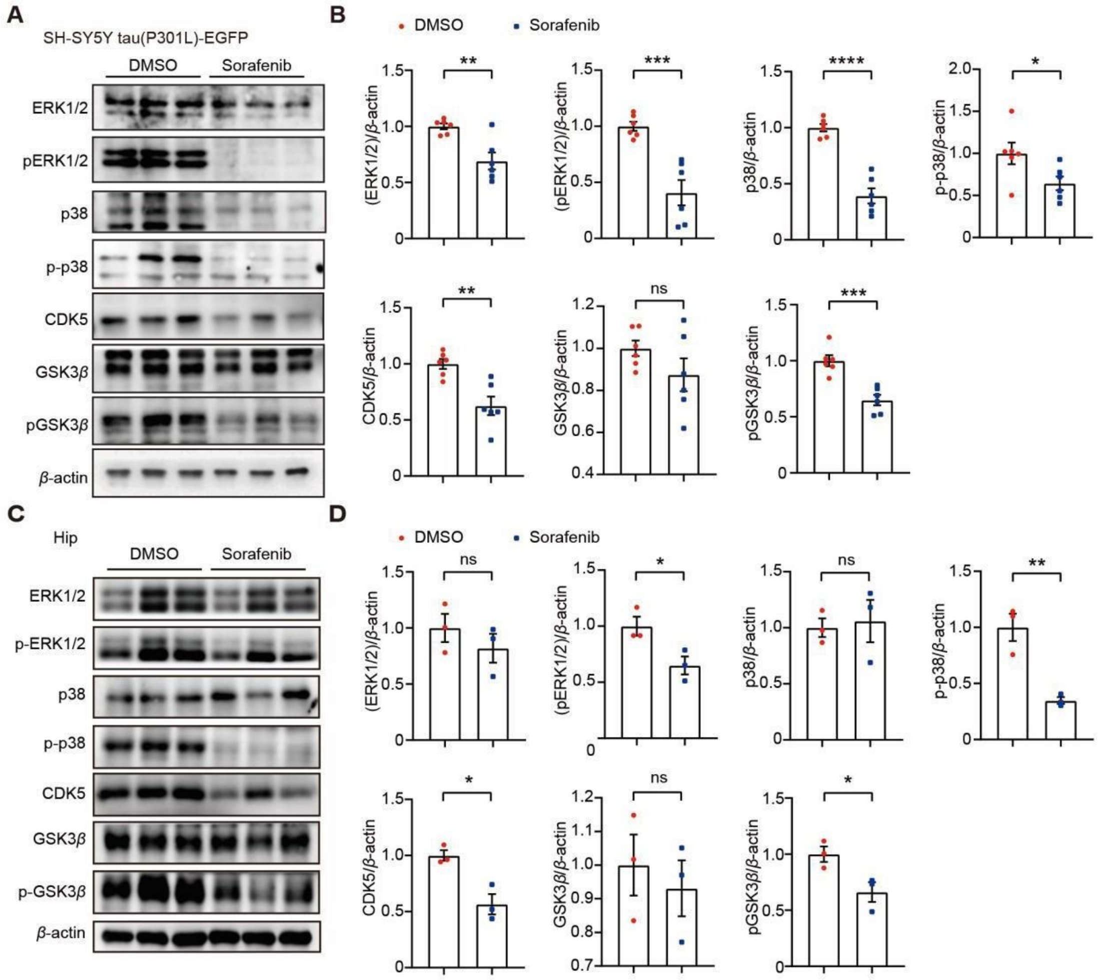

Figure S7 Sorafenib treatment inhibits multiple kinases responsible for tau phosphorylation. (A, B) SH-SY5Y tau(P301L)-EGFP cells were treated with sorafenib (10 μmol/L) or DMSO control for 24 h. Protein levels of CDK5, ERK1/2, p38, and GSK3*β* and phosphorylated forms of the latter three were analyzed by immunoblotting (A) for comparison (B). n = 6. Unpaired Student's *t*-test. (C, D) Protein levels of CDK5, ERK1/2, p38, and GSK3*β* and phosphorylated forms of the latter three in hippocampal lysates of sorafenib-treated PS19 mice and controls were analyzed by immunoblotting (C) for comparison (D). n = 3. Unpaired Student's *t*-test. Data are presented as mean ± SEM; *$P < 0.05$; **$P < 0.01$; ***$P < 0.001$; ****$P < 0.0001$; ns: not significant.

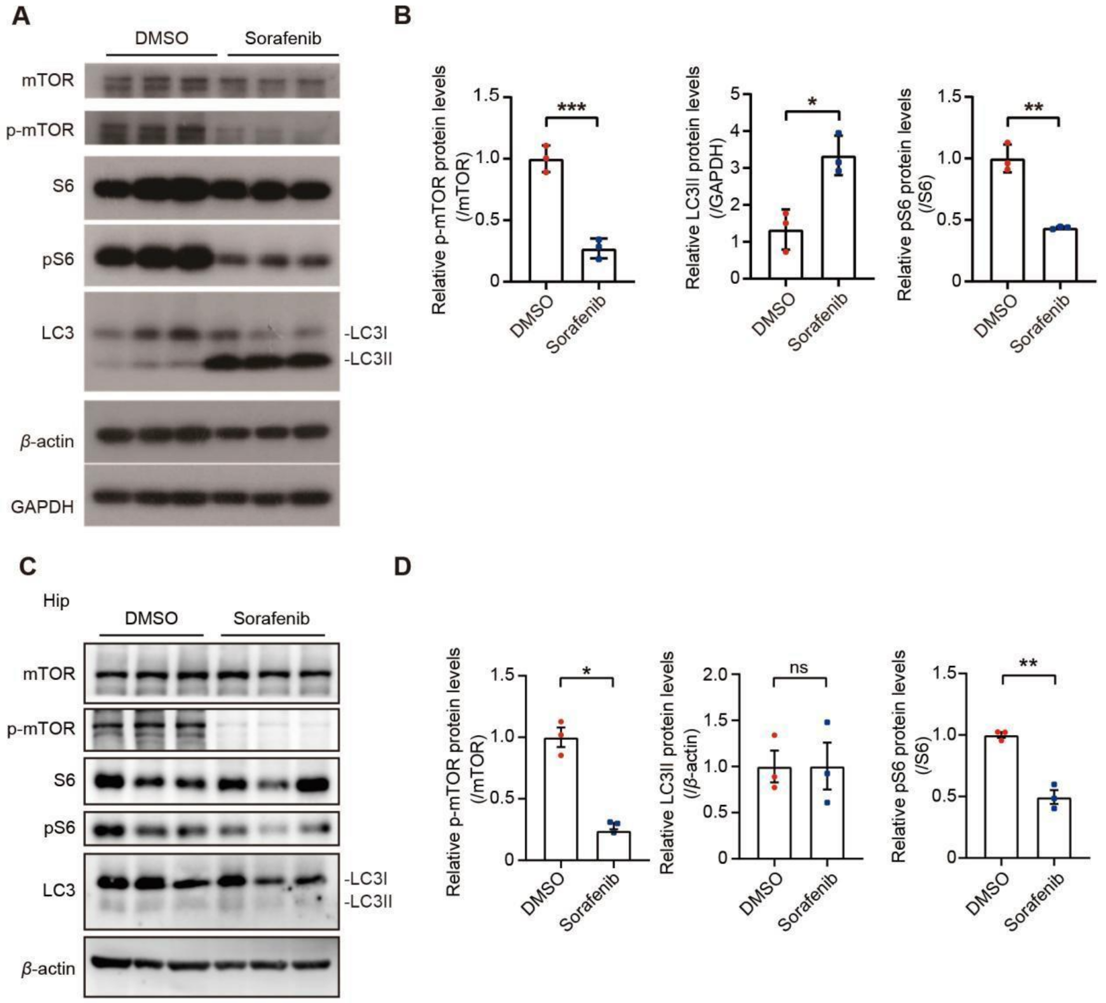

Figure S8 Sorafenib treatment promotes autophagy. (A, B) SH-SY5Y tau(P301L)-EGFP cells were treated with sorafenib (10 μmol/L) or DMSO control for 24 h. Protein levels of mTOR, phosphorylated mTOR (p-mTOR), S6, phosphorylated S6 (pS6), and LC3II were analyzed by immunoblotting (A) for comparison (B). *n* = 3. Unpaired Student's *t*-test. (C, D) Protein levels of mTOR, phosphorylated mTOR (p-mTOR), S6, phosphorylated S6 (pS6), and LC3II in hippocampal lysates of sorafenib-treated PS19 mice and controls were analyzed by immunoblotting (C) for comparison (D). *n* = 3. Unpaired Student's *t*-test. Data are presented as mean ± SEM; *$P < 0.05$; **$P < 0.01$; ***$P < 0.001$; ns: not significant.

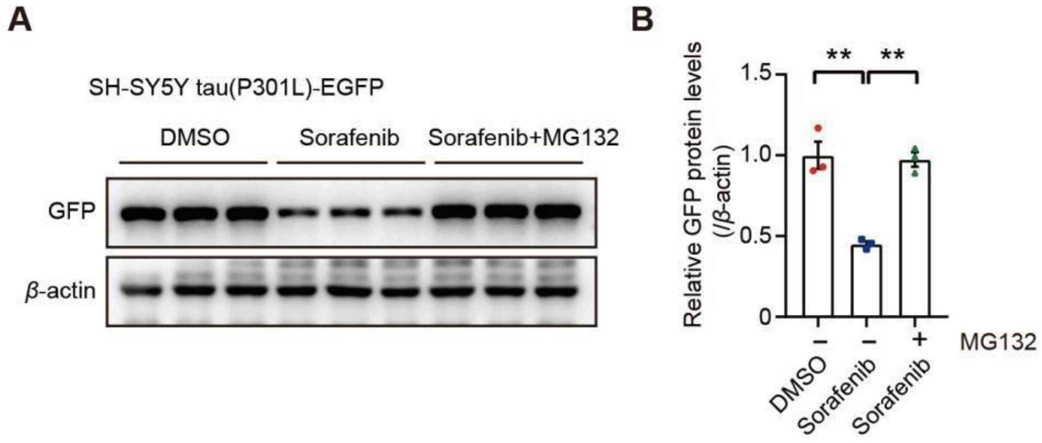

Figure S9 Sorafenib treatment promotes proteasome-mediated tau degradation. (A, B) SH-SY5Y tau(P301L)-EGFP cells were treated with sorafenib (10 μmol/L) or DMSO control for 24 h. The proteasome inhibitor MG132 (10 μmol/L) was added to the sorafenib group 10 h before sample collection. Equal amounts of protein lysates were analyzed by immunoblotting (A) for comparison (B). n = 3. One-way ANOVA with Tukey's multiple comparisons test. Data are presented as mean ± SEM; $^{**}P < 0.01$.

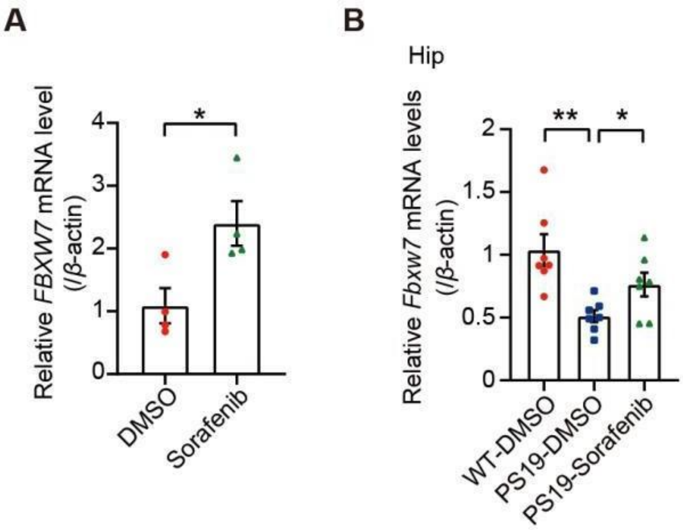

Figure S10 Sorafenib treatment promotes *FBXW7* mRNA levels. (A) SH-SY5Y tau(P301L)-EGFP cells were treated with sorafenib (10 μmol/L) or DMSO control for 24 h. The mRNA levels of *FBXW7* were analyzed by qPCR for comparison. n = 3. Unpaired Student's t-test. (B) The *Fbxw7* mRNA levels in hippocampal tissues of sorafenib-treated PS19 mice and controls were analyzed by qPCR for comparison. n = 7. One-way ANOVA with Tukey's multiple comparisons test. Data are presented as mean ± SEM; $^{*}P < 0.05$; $^{**}P < 0.01$.

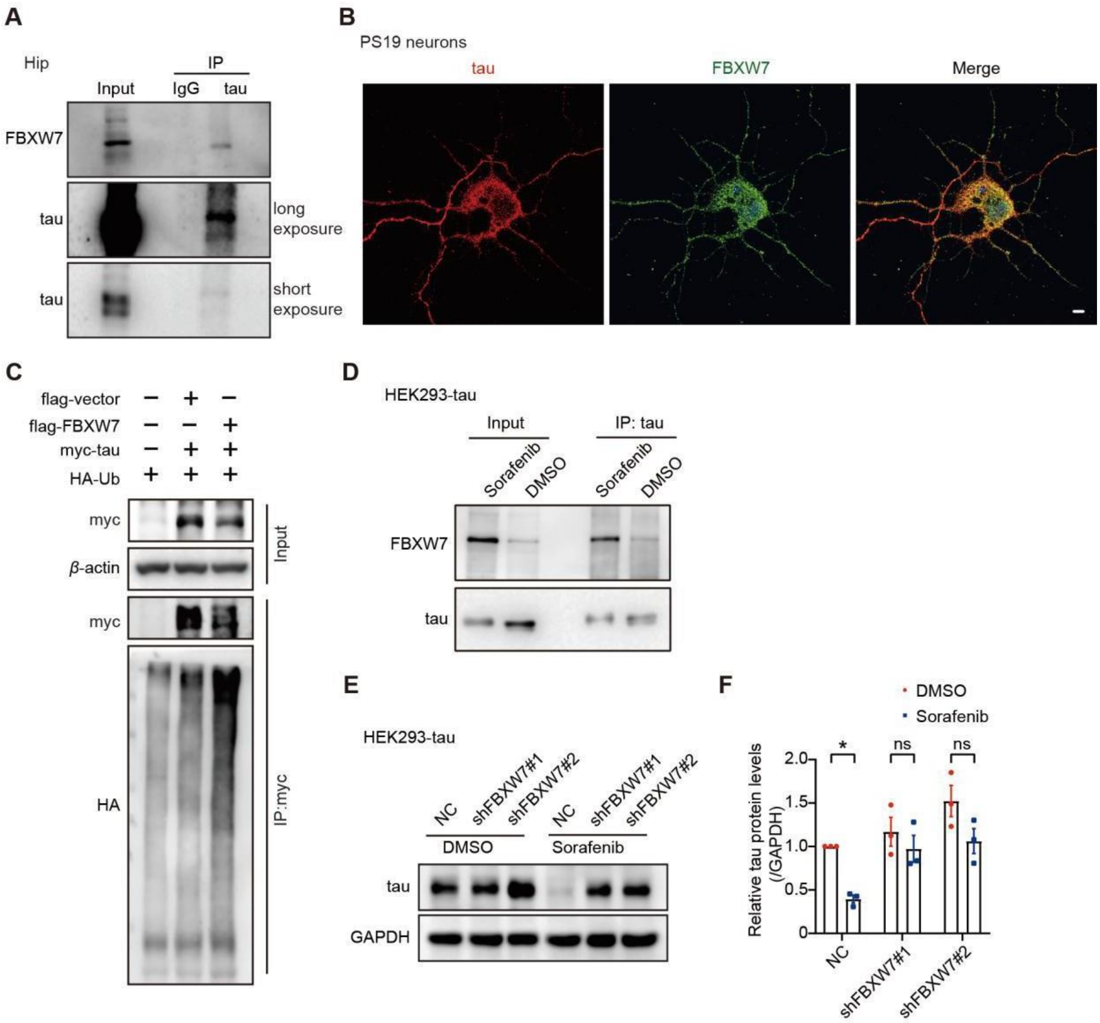

Figure S11 FBXW7 interacts with tau and facilitates tau ubiquitination. (A) Equal protein amounts of hippocampal (hip) lysates of WT mice were immunoprecipitated (IP) with an anti-tau antibody or IgG. Immunoprecipitated proteins were immunoblotted for tau and FBXW7. (B) Cultured primary neurons of PS19 mice were infected with AAV-FBXW7 for 7 days. Tau (in red) and FBXW7 (in green) were analyzed by immunofluorescence. Scale bar: 5 μm. (C) HA-Ub, myc-tau, flag vector, and flag-FBXW7 were transfected individually or collectively into HEK293T cells. Equal amounts of protein lysates were subjected to IP with an anti-myc antibody and immunoblotting with anti-myc and anti-HA antibodies. (D) HEK293 cells stably expressing tau (HEK293-tau) were treated with sorafenib (10 μmol/L) or DMSO control for 24 h. Equal amounts of cell lysates were subjected to IP with an anti-tau antibody and immunoblotting with anti-FBXW7 and anti-tau antibodies. (E, F) HEK293-tau cells were infected with lentiviruses expressing *FBXW7* shRNAs (*shFBXW7#1* and *shFBXW7#2*) for 72 h, and then treated with sorafenib (10 μmol/L) or DMSO control for 24 h. Tau protein levels were analyzed by immunoblotting (E) for

comparison (F). $n = 3$. Two-way ANOVA with Sidak's multiple comparisons test. Data are presented as mean ±SEM; $*P < 0.05$; ns: not significant

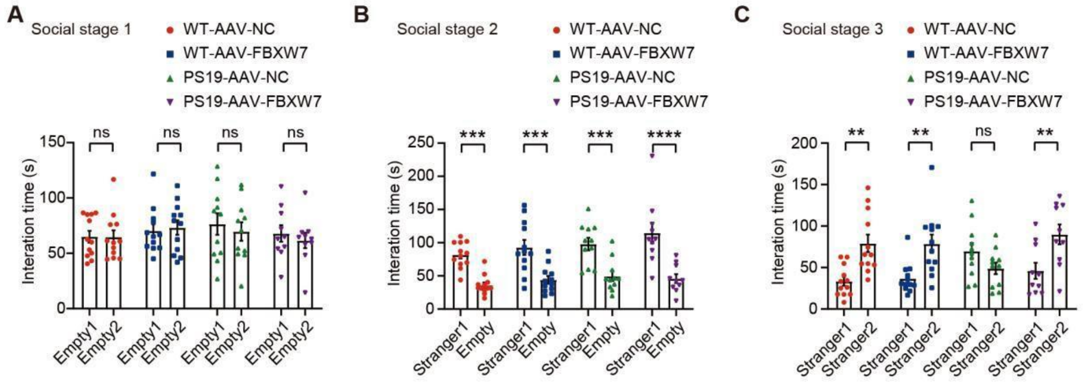

Figure S12 FBXW7 overexpression attenuates social memory in PS19 mice. (A-C) In the three-chamber social interaction test, treated mice were first studied for their time spent interacting with two empty cages (Empty1 and Empty2) in social stage 1 (A). Mice were then tested for their time spent interacting with a strange mouse (Stranger1) and with an empty cage (Empty) in social stage 2 (B). Mice were also tested for their time spent interacting with the familiar mouse (Stranger1) and with a new strange mouse (Stranger2) in social stage 3 (C). $n = 12$ mice for WT-AAV-NC, $n = 12$ mice for WT-AAV-FBXW7, $n = 11$ mice for PS19-AAV-NC, and $n = 10$ mice for PS19-AAV-FBXW7. Two-way ANOVA with Tukey's multiple comparisons test. Data are presented as mean ± SEM; $**P < 0.01$; $***P < 0.001$; $****P < 0.0001$; ns: not significant
